# Eolian erosion of polygons in the Atacama Desert as a proxy for hyper-arid environments on Earth and beyond

**DOI:** 10.1038/s41598-022-16404-y

**Published:** 2022-07-20

**Authors:** Christof Sager, Alessandro Airo, Felix L. Arens, Dirk Schulze-Makuch

**Affiliations:** 1 Astrobiology Research Group, Zentrum Für Astronomie Und Astrophysik, Technische Universität Berlin, 10623 Berlin, Germany; 2 Museum Für Naturkunde, Leibniz-Institut Für Evolutions- Und Biodiversitätsforschung, 10115 Berlin, Germany; 3 Section Geomicrobiology, German Research Centre for Geosciences (GFZ), 14473 Potsdam, Germany; 4 Department of Experimental Limnology, Leibniz-Institute of Freshwater Ecology and Inland Fisheries (IGB), 12587 Stechlin, Germany; 5 School of the Environment, Washington State University, Pullman, WA 99164 USA

**Keywords:** Geology, Geomorphology, Sedimentology

## Abstract

Polygonal networks occur on various terrestrial and extraterrestrial surfaces holding valuable information on the pedological and climatological conditions under which they develop. However, unlike periglacial polygons that are commonly used as an environmental proxy, the information that polygons in the hyper-arid Atacama Desert can provide is little understood. To promote their use as a proxy, we investigated a polygonal network within an inactive channel that exhibits uncommonly diverse surface morphologies and mineral compositions, using geochemical and remote sensing techniques. Our findings show that the polygons belong to a continuous network of the same genetic origin. Their differences result from post-formational differential eolian erosion up to 50 cm depth, exposing indurated subsurface horizons rich in sulfate or nitrate and chloride. Their location in an ancient channel could lead to the misinterpretation of fluvial polygon erosion, however, we find no such signs but evidence for aqueous resurfacing of microtopography by fog and minimal rainwater infiltration. Our findings extend the use of polygons as proxies in the Atacama Desert, indicating saline soils and hyper-arid conditions. We conclude that this example of polygon erosion can guide future polygon research, especially regarding the use of erosional surfaces on Earth and beyond to gain valuable subsurface insights.

## Introduction

Polygonal networks (PNs), a type of patterned ground, are characteristic geometric surface features that occur in terrestrial landscapes of varying climates^1,2^ and on other planetary bodies^3–5^. They are formed by a multitude of mechanisms including desiccation^1^, thermal contraction^6^, sublimation^3^, freeze–thaw cycles^7^ among others. At the surface, PNs can be recognized by the downward extending wedges, which are filled with ice and/or sand that visibly outline the polygons, or by the polygon morphology where the center can be elevated (high-center), level (flat-center), or depressed (low-center) compared to the surrounding wedges. PNs exhibit varying pattern geometries, determined by the angles at which the wedges intersect, where hexagonal (120°, three-ray intersection) and orthogonal (90°, four-ray intersection) are most common. The development of PNs is largely controlled by local morphology, ground-, climate-, and moisture conditions^2^. These interactions are the basis for using PNs as a proxy for information on the environmental setting such as local soil type and climatic conditions. However, such application is currently limited to periglacial areas on Earth and Mars, where PNs are most widespread and have been studied intensively for more than a century^4,5,8,9^. In particular, polygon morphology can indicate substrate difference, as e.g., high-center sublimation polygons develop in debris-covered glacier ice while low-relief sand wedge polygons form in ice-cemented soil^10^. The size of polygons can indicate the depth of crack propagation, while polygon morphology and type can be used to indicate the different polar climate zones in the Antarctic Dry Valleys, e.g., with high-center sublimation polygons in the more cold upland areas and flat-center ice-wedge polygons near the warmer coastal areas^5,10^. Furthermore, PNs can indicate climate shifts, e.g., the degradation of ice wedges due to an increase in permafrost-ground temperature^11^.

However, the processes controlling the formation and development of polygons in periglacial regions cannot be directly applied to PNs occurring in the temperate and hyper-arid Atacama Desert^12–14^; consequently, their use as a proxy is likely to be different as well. PNs in the Atacama Desert exhibit significant differences, especially because of their soil cementation by salt instead of ice, the lack of enduring freezing temperatures, and more restricted availability of water. Although many studies investigated the climatological and pedological evolution of the Atacama Desert in the last decades^12–18^, the use of PNs as an environmental proxy for climate and soil type has not been realized in this context.

The Atacama Desert is particularly well suited for studying the use of PNs as a proxy for long-term environmental conditions because it represents the oldest non-polar desert and exhibits hyper-aridity over the last ~ 12 Ma^19^, intercepted by stages of semi-arid to arid climate^18^. The low precipitation rates have resulted in minimal fluvial washout but the massive accumulation of dust and salts such as sulfates that often overlie chloride and nitrate horizons, which are cementing the upper meters of the soil^12,13,20–22^. This regionally ubiquitous soil is located on inactive and morphologically stable alluvial deposits^23^, and has been classified into different locally termed horizons, which can vary in degree of development and thickness^21^: The upper ~ 30 cm are called *chusca*, which is a gypsic horizon, i.e., a non-indurated gypsum-rich horizon, that consists of a few centimeter thick layer of loose sediment containing palm-sized gypsum-anhydrite aggregates termed *losas* underlain by a *vesicular horizon* that is composed of powdery, and porous gypsum and anhydrite. Below lies the *costra,* a petrogypsic horizon, i.e., indurated gypsum-rich horizon, consisting of sandy-to-gravelly sediment that is intensely cemented by low soluble sulfates (gypsum, anhydrite and bassanite) and can extend up to a thickness of 2 m. Underneath the *costra* horizon is the *caliche,* a petrosalic horizon, i.e., a horizon indurated by salts more soluble than gypsum^24^, which are in this case mainly nitrates and chlorides, extending up to a thickness of 5 m.

**Figure 1:**
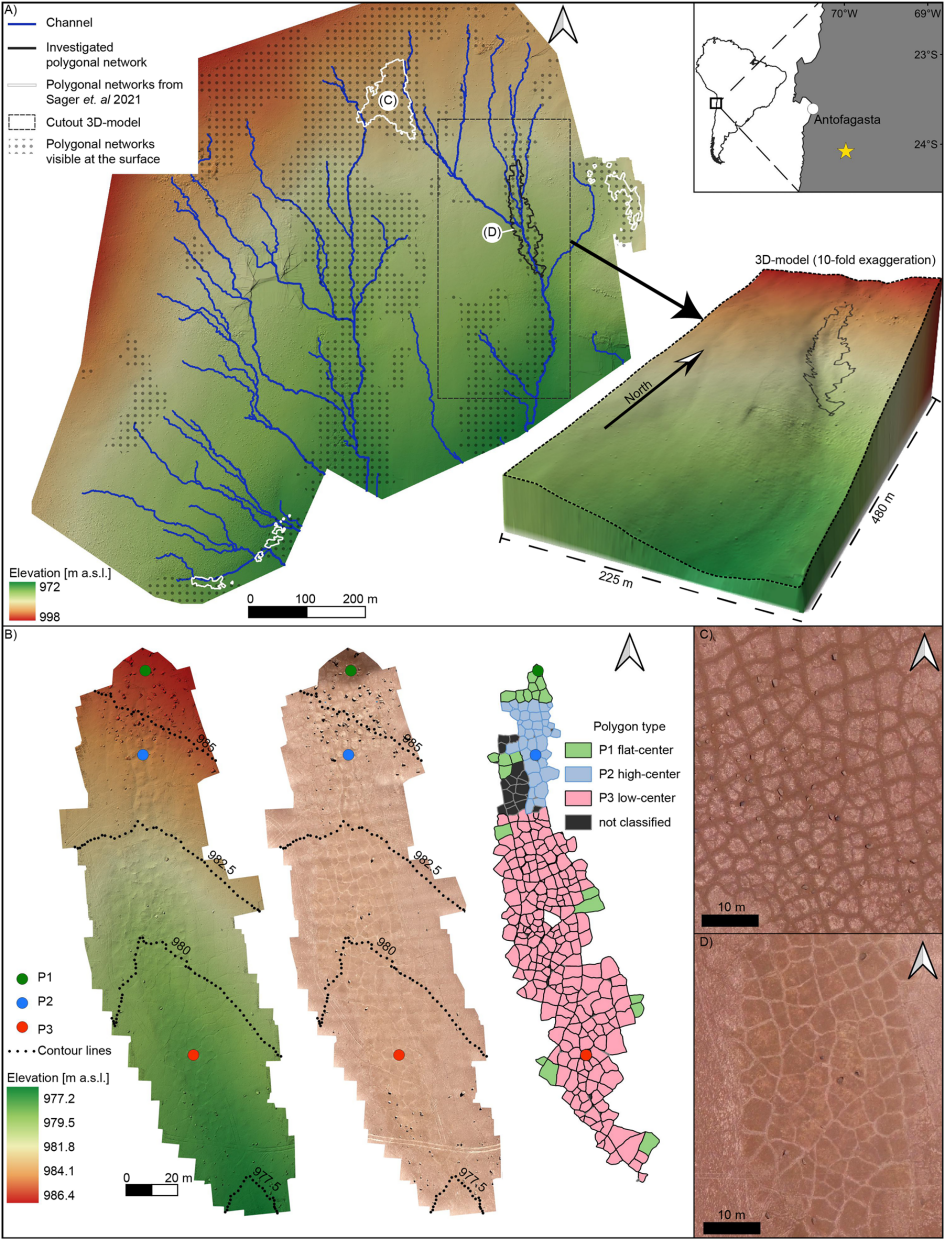
Color shaded digital elevation models (DEMs) and orthophotos of the study area. (**A**) The upper right box shows a location map of the study site in South America and near Antofagasta, Chile (star symbol). The DEM (overview model) shows the surrounding terrain on which the investigated polygon network (PN) is located. The 3D-model (lower right corner) with tenfold vertical exaggeration shows the PN in an inactive channel. The letters on white background show the location of Fig. 1 C,D. (**B**) Maps of the close-up model show the PN with sampling locations (P1﻿: − 24.07846, − 69.99319, P2: − 24.07741, − 69.99338, P3: − 24.07711, − 69.99337) as a shaded DEM, as an orthophoto, and the polygon classification. (**C**) Orthophoto of a non-eroded PN outlined by dark sand wedges, while at (**D**) the polygonal pattern in P3 is inverted due to eolian erosion with polygons that appear dark compared to their sand wedges. Elevation is given in meter above sea level (m a.s.l.).

PNs occurring on these soils in the Atacama Desert (Fig. 1) exhibit ~ 1 m deep and salt-poor sand wedges outlining salt-rich polygons of a uniform flat-center morphology^25^. They exhibit an orthogonal to hexagonal pattern geometry, and elongated polygons have either one or two dominant orientations, preferably parallel or perpendicular to the slope. However, the details on the formation of PNs remain under debate, such as the driving forces behind the crack formation. The proposed processes range from thermal contraction, where cementation by salts results in cohesive soils and makes them susceptible to thermal stress^25^ and/or salt dehydration^12,14^, both being cyclic processes. The details on the importance of rain events for PN formation remain unclear, although, its involvement is certain as rainwater infiltration, which is insufficient to fully wash out the salts, creates the vertical salt sequence of these indurated soils^12,22^. To evaluate the use of PNs as a proxy for climatological and soil conditions, we investigated a 200 m long PN that is exceptional in that it displays multiple and novel morphological and geochemical variations.

## Results

**Figure 2:**
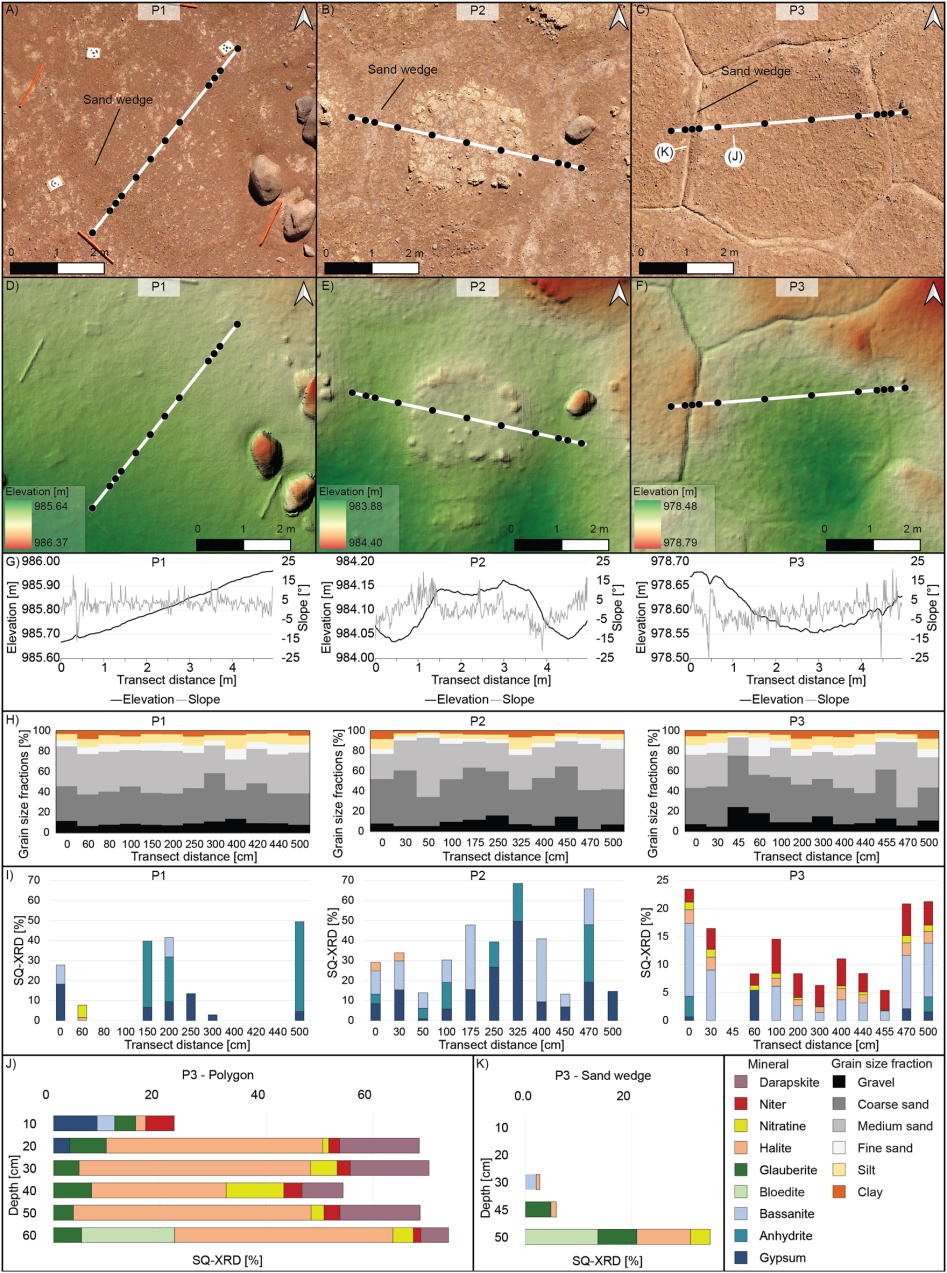
Morphological and geochemical data of the study site. (**A**, **B**, **C**) The polygons (P1, P2, P3) and sample locations (black dots) along the transect (white line) displayed as orthophotos, (**D**, **E**, **F**) digital elevation models, (**G**) polygons profiles, (**H**) grain size distribution and (**I**) salt composition based on semi-quantitative X-ray diffraction (SQ-XRD) along the soil transects and (**J**, **K**) two depth profiles for the polygon and the sand wedge at P3. Elevation is given in meter above sea level (m a.s.l.).

The morphological and geometrical characterization of the investigated PN and its surrounding was based on two drone-based digital elevation models and orthophotos here referred to as the overview model (Fig. 1A) and close-up model (Fig. 1B). The sedimentary and geochemical characterization of three surface transects and two depths profiles was done through field observations and sample analysis of their soluble content, grain size distribution, and mineralogical composition using X-ray diffraction (Fig. 2). The investigated PN is located within an elongated depression of an alluvial fan, however, only the drone-based topography reconstruction and channel modeling show clearly that the PN lies within an inactive channel (Fig. 1A). The surface of the PN is littered with gravel and boulders, while sand dominates the upper 10 cm (Fig. 2H). The polygons share a similar clastic mineralogy dominated by quartz, feldspars, and minor amounts of amphiboles (Supplementary Fig. S3). All polygons show a relatively similar size with a mean diameter of 3.9 ± 1.7 m and exhibit connected sand wedges forming a continuous network with a primarily hexagonal pattern geometry with two main polygon orientations to the NS and EW (Supplementary Fig. S1), consistent with a uniform and simultaneous formation for the entire PN.

**Figure 3:**
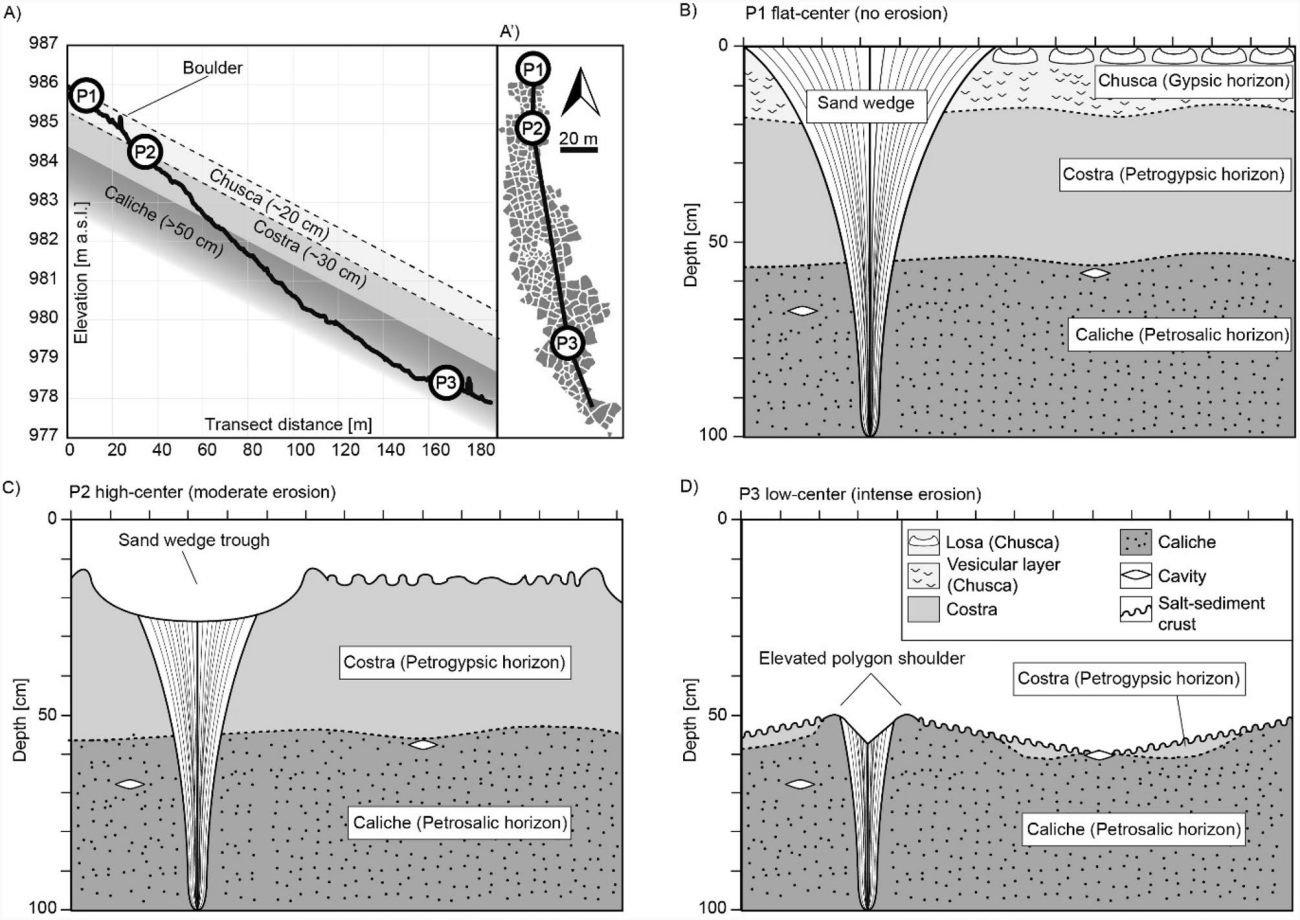
Conceptual interpretation of polygonal network (PN) erosion in the Atacama Desert. (**A**) Slope profile (black line) along the PN with sample locations (P1, P2, P3) and the common soil horizons (schematic illustration, not to scale). (A’) The profile position (black line) along the PN (grey). (**B**) The flat-center P1 polygons represent non-eroded polygons; modified from ^25^ (**C**) The high-center P2 polygons with moderate erosion (0.2 m) exposing the *costra* horizon. (**D**) The low-center P3 polygons with intense erosion (0.5 m) exposing the deeper *caliche* horizon.

As described above, the PN exhibits similarities regarding geometry, orientation, grain size distribution, and mineralogy of the non-soluble fraction (Fig. 2H, Supplementary Fig. S1, Supplementary Fig. S3). However, we distinguish three polygon types (P1, P2, P3) within this network based on variations in surface morphology and mineralogy of the cementing salts (Figs. 1, 2 and 3): The P1 polygons located upslope of the network, show a flat-center morphology and are outlined by dark varnished and broad sand wedges (Fig. 2), analogous to the common polygons which are visually recognizable on 28% of the reconstructed surrounding terrain of 0.73 km^2^ (Fig. 1). Their surface exhibits salt-poor sediment and embedded *losas* (palm-sized gypsum and anhydrite aggregates), which is typical for the upper part of the *chusca* horizon (gypsic horizon) (Fig. 2). The P2 polygons located up to mid-slope show a high-center morphology with depressed and broad sand wedges (Fig. 2). In contrast to the P1 surface, the surface of the elevated P2 polygons lacks the loose sediment cover, is more indurated, and exhibits a high average content of soluble salts of 36% along the polygon transect dominated by sulfates (gypsum and anhydrite), which is a soil build-up similar to the *costra* horizon (petrogypsic horizon). The P3 polygons located mid- to downslope show a low-center morphology with narrow incised sand wedges bound by elevated polygon shoulders (Fig. 2, Supplementary Fig. S5). The polygon is composed of a mix of salts of high solubility (halite, niter, and nitratine) and low solubility (gypsum, bassanite, and anhydrite), which is a soil composition similar to the upper part of the *caliche* horizon (Fig. 2). The polygon surface exhibits domed salt-sediment crusts and cavities (Fig. 3, Supplementary Fig. S5). The salt content, mainly halite, nitratine, and darapskite, increases up to 74% towards the base of the polygon profile at a depth of 60 cm (Fig. 2, Supplementary Fig. S2). The investigated sand wedge contains less salts than the polygons but shows a similar increase in salt content with depth, reaching 35% at its base at 50 cm depths.

A central observation is that the downslope sequence of the exposed polygon surfaces (P1, P2, P3) corresponds to the vertical sequence of soil horizons with *chusca*, *costra,* and *caliche* (Fig. 3). In particular, the P1 polygons show no signs of erosion and resemble the common surface horizon (*chusca*, gypsic horizon) in the surrounding. The two other polygon types (P2 and P3) show no signs of fluvial erosion but clear signs of eolian erosion. We interpret the exposed sulfate-cemented surface at P2 as the *costra* horizon (petrogypsic horizon), a horizon below the *chusca.* The relief at P2 with a high-center morphology and broad troughs above sand wedge is consistent with eolian erosion, whereby the less cemented sand wedges are more intensely eroded than the cemented polygon center. We interpret the nitrate- and chloride-containing surface at P3 as the *caliche* horizon (petrosalic horizon) which underlies the *costra*, exposed by additional surface erosion (Fig. 3). We estimate an eolian erosion of ~ 50 cm on P3 surfaces, based on the lack of a *chusca* and *costra* horizon, which have a thickness of 20 and 30 cm, respectively, in a soil pit in a proximity of 200 m^25^ (Fig. 3). This erosional depth is supported by the observation that the sand wedge at P3 is 50 cm deep, while non-eroded sand wedges in the nearby soil pit have a depth of 100 cm. Further indication for eolian erosion is the observed decrease in sand wedge width from P1 to P3 polygons, while polygon size and geometry are similar. We interpret this decrease in sand wedge width to result from the erosion of the upper wider parts of the sand wedges exposing their narrower base. The eolian erosion being caused by the strong westerly winds in the Yungay valley^16^ is supported by the preferential erosion of the windward sides (west-facing) of the sand wedge troughs (Supplementary Fig. S4) and abundant scour marks around the base of boulders (Supplementary Fig. S5).

Further evidence for the eolian erosion of a preexisting PN is the relation between slope and polygon alignment. Comparing the alignment of the polygons with the average slope direction of the surrounding area (250 × 400 m, dashed box in Fig. 1A) results in a perpendicular or parallel slope alignment (Supplementary Fig. S1). Such a relation is well known from periglacial polygons^2^ and has also been shown for PNs in the vicinity of the study area^25^. However, this alignment is lost when comparing the polygon orientation with the slope direction of each individual polygon surface using the close-up model (Supplementary Fig. S1). This deviation is consistent with differential eolian surface erosion changing the slope direction but not polygon orientation. Besides changes in morphology and geochemistry, the erosion led to a “pattern inversion” as the common flat-center polygons appear bright and are outlined by dark sand wedges, whereas at P3 surfaces the polygon interior is darker than their narrow sand wedges (Fig. 1). This shift to a darker polygon color is probably due to the removal of the bright sulfates. The darker appearance was intensified when the hygroscopic salt crusts at P3 became wetted by deliquescence during periods of high air humidity (Supplementary Fig. S5)^26^. Such cycles of minimal moisture input due to dew and fog probably resulted in the observed clast encrustation by salts and undulating and domed salt-sediment crusts (Supplementary Fig. S5). At the same time, downward leaching by rare rain events is indicated by the lower halite content in the first 10 cm at P3, compared to depths below 10 cm (Fig. 2). These rain events may have further modulated the salt crusts because ephemeral salt efflorescence has been observed on the surface of many soils after the recent rain events^14^.

## Discussion

### Development and erosional history of the polygonal network

The eolian erosion of the here investigated PN resulted in the exposure of subsurface horizons, which allows for the possibility to acquire additional information particularly through remote sensing if no in situ field observation is possible. However, only using remote sensing data for interpretation without in situ field observations and subsequent sample analysis, can lead to misinterpreting the lateral variations in morphology and composition to result from fluvial erosion or different formation processes. Based on our findings in the context of the established knowledge on the past climate and soil formation processes in the Atacama Desert, we propose the following developmental history for the here studied PN. The alluvial fan is the host material of the PN and at most of Miocene age^27^. The channel activity in the study area probably ceased in the Late Pliocene to Early Pleistocene and was followed by reduced precipitation and massive salt accumulation^13^. Consequently, we assume that PN formation, continuing until today, began after the Late Pliocene (~ 2.6 Ma) and the incision of the channel but probably required first a sufficient accumulation and cementation by salts for initiation^12,13^. These salts, indurating the soil are a prerequisite for polygon development, presumably due to dehydration of sulfates^12,13^ and/or due to thermal contraction in salt-cemented ground^25^. Furthermore, the presence of highly soluble salts at shallow depth indicates an incomplete washout as a result of a predominantly hyper-arid climate punctuated by rare rain events, as suggested before^12–14,22^. Hence, the relationship between the high soluble salt content and hyper-arid conditions on the one hand and the presence of PNs that developed under these conditions, on the other hand, allows for the use of PNs as a proxy for saline soils (pedological condition) and predominantly hyper-arid conditions. Advantageously, PNs can be frequently detected with remote sensing using e.g., satellite imagery, which makes them a suitable environmental proxy at the regional scale. However, their surficial detection can be hindered by sediment covering, e.g., by boulder fields or eolian deposits controlled by local wind pattern^22^. Thus, the inability to observe PNs neither necessarily implies that a polygonal ground is lacking nor that saline soils and hyper-arid conditions are absent.

### Insights from eroded polygonal networks

The differential erosion of PNs can provide further insights into surface and subsurface characteristics: (1) The exposure of the subsurface geometry of wedges can be used to deduce the stress regime, the cracking depth and cementation depth. Yet, the quantification of the sand wedge geometry based on aerial imagery is not always possible in the visible spectrum and could be aided by e.g., spectral imaging highlighting material differences. (2) Differences in polygon morphology can indicate variations in sediment induration and therefore resistance to eolian erosion, e.g., the development of a high-center morphology at P2 surfaces is indicative of less cemented sand wedges compared to the elevated polygon center. Further, the relief development of elevated polygon shoulders, e.g., at P3 surfaces may point to salt-related ground deformation, for example, due to the thermal expansion of the now exposed highly soluble salts during ground heating, also known from periglacial polygons^28^ or volumetric expansion based on the hydration of sulfates after moisture input. (3) The surficial exposure of highly soluble salts at P3 provides information on the compositional stratification of the soils, which in our case results in a pattern inversion from bright polygons outlined by dark sand wedges (P1) to dark polygons with surface crusts outlined by bright sand wedges (P3). Furthermore, the presence of these crusts can provide insights on the rates of concurrent processes continuously reshaping the soils. While on the one hand eolian erosion promotes the exposure of the soluble subsurface salts, on the other hand rain events will inevitably transport the salts back into the subsurface. Hence, we can conclude, that the rate of eolian erosion has been far more rapid than the down washing of the salts. However, the occurrence of surface crusts indicates surficial alteration that is more rapid than eolian erosion. Presumably the surface crusts have been created by minor rain events with minimal soil infiltration and fog that frequently occurs up to 90 km inland and below elevation of 1000–1300 m a.s.l.^22,29,30^. (4) The depth-related sequence of salts of different solubility can be used to infer the dominating water pathways (rain vs. groundwater), as is the case for this PN where the solubility increases with depth corresponding to a downward migration of rainwater, which is common for alluvial surfaces in this area^12,22^. Interestingly, recent unusually frequent rain events, as recorded in 2015 and 2017, have been linked to climate change^31^; such an increase in precipitation will eventually washout the near-surface salts and inevitably change the PNs, which thus can serve as a proxy for climate change. (5) Lastly, our study contributes to a better understanding of the dust sources and sinks in the Atacama Desert, as we show that the saline subsurface material of PNs can at least be locally remobilized through eolian erosion and introduced into the dust cycle^18^.

Given this applicability, differential erosion of PNs is of particular value in remote locations where field sampling is not always possible but remote sensing can be applied. Hence, our findings can serve as a reference example and guideline for PN evaluation in the Atacama Desert and in other arid environments affected by dry erosion, such as the Gobi Desert^32^, the Dry Valleys of Antarctica^33,34^, and on Mars^5,35^.

### Implications for Martian polygons

Mars is vastly covered by PNs with the majority being interpreted as periglacial features situated near polar regions and characterized by their ice cementation and cryogenic origin^5,36^. Further, Mars exhibits putative desiccation polygons^37,38^, which often occur in regions with sulfate- or chloride-containing soils such as the southern Martian highlands^39,40^. Lateral changes in PN morphology and/or composition have also been observed on Mars, e.g., in Utopia Planitia where low-center polygons occur in a topographic low and transition further upslope into high-center polygons that become flat accompanied by a widening of their sand wedge troughs^35^. Although this depression is altered by sublimation and margin collapse, the morphological transition is strikingly similar to our findings in the Atacama Desert. However, the Martian low-center polygons were interpreted to be aggrading and the high-center polygons degrading, whereas in this study these types were both degrading due to wind erosion. Based on the observations in the Atacama Desert, we think that also low-center polygons can have not only aggrading but also degrading wedges. Hence, we suggest that wind erosion, known to be a dominant modifier of the Martian surface^41^, should be considered when evaluating the PNs on Mars or elsewhere. Lastly, thermal contraction and sulfate dehydration, which are both processes proposed to play a role for PN development in the Atacama Desert could be relevant for polygon development on Mars as well. In particular, polygons in Meridiani Planum bear similarities to sulfate-containing polygons in the Atacama Desert and have been interpreted to result from wetting/drying cycles causing sulfate re-/dehydration^42^, which could have implications for their potential habitability. Additionally, sulfate^40^ and/or chloride-containing ground^39^ would allow for thermal contraction cracking due to salt cementation, which would be an additional mode to the common thermal contraction in ice-cemented material^5,43^.

## Conclusion

We investigated a polygonal network (PN) where eolian erosion of flat-center polygons resulted in high-center and low-center polygons and the exposure of subsurface horizons with varying geochemistry. In general, PNs develop in saline soils under hyper-arid conditions in the Atacama Desert, promoting them as a proxy for these conditions. Consequently, a shift to moister conditions would be reflected in the PNs making them a valuable marker for climate change. In particular, eroded PNs provide a window into the subsurface revealing further insights regarding ground conditions and development. Hence, PNs affected by erosion need to be considered for the assessment of polygons in hyper-arid environments, in particular on Mars, where the wind is a dominant surface modifier and PNs frequently occur on saline ground. Future polygon monitoring and research, investigating their cracking mechanisms, formation rates, and occurrence in the Atacama Desert are greatly needed, to better understand patterned ground formation in non-polar areas and the entirety of proxy information they can provide.

## Methods

The sedimentological, geochemical, and morphological properties of the PN (− 24.07796, − 69.99339) were determined as follows. Three 5 m long soil transects of each polygon type (P1: 12 samples, P2: 11 samples, P3: 12 samples), stretching across a single polygon and its neighboring polygon shoulders were sampled in a depth of 0–10 cm for grain size distribution, X-ray diffraction (XRD) and salt content. Additionally, two excavated profiles at P3 along the polygon (6 samples) to a depth of 60 cm, and the sand wedge (5 samples) to a depth of 50 cm were sampled in ~ 10 cm increments. Drone flights, 3D-spatial data generation, geochemical, sedimentological, and morphological analyses were conducted using similar methods as described by Sager et al.^25^. Agisoft Metashape was used for photogrammetric reconstructions to generate digital elevation models (DEM) and orthophotos^44^. Two models were generated, here referred to as the “overview model”, which displays the PN and the surrounding topography of the alluvial fan, while the high-resolution "close-up model” displays solely the PN. Polygon mapping, analysis, and map generation were conducted with QGIS^45^. Orientational data was plotted using GeoRose^46^. For grain size analysis, samples were leached with de-ionized water and wet sieved. The salt content was calculated based on the weight difference of samples before and after leaching. For XRD analysis, dry-grinded samples and a Bruker D2 Phaser benchtop diffractometer were used, and semi-quantitative (SQ) analysis was carried out with the “Diffrac.eva” software and the “Powder Diffraction File Minerals 2019″ (database of the *international centre for diffraction data*) to determine the mineral content. The accuracy of the determination of the total soluble content based on weight differences after leaching is higher compared to the salt content based on SQ-XRD. Therefore, we calibrated the relative abundances of the identified mineral assemblages to the total salt content determined by sample leaching. Detailed methods are provided in the Appendix (Supplementary File 1).

## Supplementary Information

Supplementary Information 1.

Supplementary Information 2.

Supplementary Information 3.

## Data Availability

The authors declare that all the data supporting the findings of this study are available within the article and its Supplementary Information file, or available from the corresponding authors on request.

## References

[1] Neal JT, Langer AM, Kerr PF (1968). Giant Desiccation Polygons of Great Basin Playas. Geol. Soc. Am. Bull..

[2] Ulrich M, Hauber E, Herzschuh U, Härtel S, Schirrmeister L (2011). Polygon pattern geomorphometry on Svalbard (Norway) and western Utopia Planitia (Mars) using high-resolution stereo remote-sensing data. Geomorphology.

[3] Morison A, Labrosse S, Choblet G (2021). Sublimation-driven convection in Sputnik Planitia on Pluto. Nature.

[4] El-Maarry MR, Pommerol A, Thomas N (2013). Analysis of polygonal cracking patterns in chloride-bearing terrains on Mars: Indicators of ancient playa settings. J. Geophys. Res. Planets.

[5] Levy JS, Marchant DR, Head JW (2010). Thermal contraction crack polygons on Mars: A synthesis from HiRISE, Phoenix, and terrestrial analog studies. Icarus.

[6] Lachenbruch, A. H. in *70: Mechanics of Thermal Contraction Cracks and Ice-Wedge Polygons in Permafrost* (Geological Society of America, 1962). pp. 1–66.

[7] Kessler MA, Werner BT (2003). Self-organization of sorted patterned ground. Science.

[8] Washburn AL (1956). Classification of patterned ground and review of suggested origins. Geol. Soc. Am. Bull..

[9] Washburn, A. L. *Geocryology. A survey of periglacial processes and environments.* 2nd ed. (Arnold, 1979).

[10] Marchant DR, Head JW (2007). Antarctic dry valleys: Microclimate zonation, variable geomorphic processes, and implications for assessing climate change on Mars. Icarus.

[11] Jorgenson, M. T., Shur, Y. L. & Pullman, E. R. Abrupt increase in permafrost degradation in Arctic Alaska. *Geophys. Res. Lett.* **33** (2006).

[12] Ewing SA (2006). A threshold in soil formation at Earth’s arid–hyperarid transition. Geochim. Cosmochim. Acta.

[13] Amundson R (2012). Geomorphologic evidence for the late Pliocene onset of hyperaridity in the Atacama Desert. Geol. Soc. Am. Bull..

[14] Pfeiffer M (2021). Century scale rainfall in the absolute Atacama Desert: Landscape response and implications for past and future rainfall. Quatern. Sci. Rev..

[15] Ritter B (2019). Climatic fluctuations in the hyperarid core of the Atacama Desert during the past 215 ka. Sci. Rep..

[16] McKay CP (2003). Temperature and moisture conditions for life in the extreme arid region of the Atacama desert: Four years of observations including the El Niño of 1997–1998. Astrobiology.

[17] Schulze-Makuch D (2018). Transitory microbial habitat in the hyperarid Atacama Desert. Proc. Natl. Acad. Sci. U.S.A..

[18] Arenas-Díaz F (2022). Dust and aerosols in the Atacama Desert. Earth Sci. Rev..

[19] Jordan TE, Kirk-Lawlor NE, Blanco NP, Rech JA, Cosentino NJ (2014). Landscape modification in response to repeated onset of hyperarid paleoclimate states since 14 Ma, Atacama Desert, Chile. Geol. Soc. Am. Bull..

[20] Michalski G, Böhlke JK, Thiemens M (2004). Long term atmospheric deposition as the source of nitrate and other salts in the Atacama Desert, Chile: New evidence from mass-independent oxygen isotopic compositions. Geochim. Cosmochim. Acta.

[21] Ericksen GE (1983). The Chilean Nitrate Deposits: The origin of the Chilean nitrate deposits, which contain a unique group of saline minerals, has provoked lively discussion for more than 100 years. Am. Sci..

[22] Arens FL (2021). Geochemical proxies for water-soil interactions in the hyperarid Atacama Desert, Chile. CATENA.

[23] Nishiizumi K, Caffee MW, Finkel RC, Brimhall G, Mote T (2005). Remnants of a fossil alluvial fan landscape of Miocene age in the Atacama Desert of northern Chile using cosmogenic nuclide exposure age dating. Earth Planet. Sci. Lett..

[24] Finstad K, Pfeiffer M, Amundson R (2014). Hyperarid Soils and the Soil Taxonomy. Soil Sci. Soc. Am. J..

[25] Sager C, Airo A, Arens FL, Schulze-Makuch D (2021). New type of sand wedge polygons in the salt cemented soils of the hyper-arid Atacama Desert. Geomorphology.

[26] Davila AF, Hawes I, Ascaso C, Wierzchos J (2013). Salt deliquescence drives photosynthesis in the hyperarid Atacama Desert. Environ. Microbiol. Rep..

[27] SERNAGEOMIN. *Mapa Geológico de Chile: versión digital. Base Geologica.* 4th ed. (Servicio Nacional de Geología y Minería, 2003).

[28] Murton JB, Worsley P, Gozdzik J (2000). Sand veins and wedges in cold aeolian environments. Quatern. Sci. Rev..

[29] Rech JA, Quade J, Hart WS (2003). Isotopic evidence for the source of Ca and S in soil gypsum, anhydrite and calcite in the Atacama Desert, Chile. Geochimica et Cosmochimica Acta.

[30] del Río C (2018). ENSO influence on coastal fog-water yield in the Atacama Desert, Chile. Aerosol Air Qual. Res..

[31] Azua-Bustos, A. & G. Fairén, A. *The effects of climate change on the Atacama Desert as a pertinent Mars analog model* (2020).

[32] Li H (2014). A new sand-wedge–forming mechanism in an extra-arid area. Geomorphology.

[33] Sletten, R. S. Resurfacing time of terrestrial surfaces by the formation and maturation of polygonal patterned ground. *J. Geophys. Res.* **108** (2003).

[34] Bockheim JG, Kurz MD, Soule SA, Burke A (2009). Genesis of active sand-filled polygons in lower and central Beacon Valley, Antarctica. Permafrost Periglac. Process..

[35] Séjourné A (2011). Scalloped depressions and small-sized polygons in western Utopia Planitia, Mars: A new formation hypothesis. Planet. Space Sci..

[36] Washburn, A. L. *Periglacial processes and environments* (Arnold, 1973).

[37] El-Maarry MR (2014). Potential desiccation cracks on Mars: A synthesis from modeling, analogue-field studies, and global observations. Icarus.

[38] Stein N (2018). Desiccation cracks provide evidence of lake drying on Mars, Sutton Island member, Murray formation Gale Crater. Geology.

[39] Osterloo, M. M., Anderson, F. S., Hamilton, V. E. & Hynek, B. M. Geologic context of proposed chloride-bearing materials on Mars. *J. Geophys. Res.* **115** (2010).

[40] Bishop, J. L. *et al.* Mineralogy of Juventae Chasma: Sulfates in the light-toned mounds, mafic minerals in the bedrock, and hydrated silica and hydroxylated ferric sulfate on the plateau. *J. Geophys. Res.* **114** (2009).

[41] Grant, J. A. *et al.* Degradation of Victoria crater, Mars. *J. Geophys. Res.* **113** (2008).

[42] Amundson R (2018). Meteoric water alteration of soil and landscapes at Meridiani Planum Mars. Earth Planet. Sci. Lett..

[43] Brooker LM (2018). Clastic polygonal networks around Lyot crater, Mars: Possible formation mechanisms from morphometric analysis. Icarus.

[44] AgiSoft. *Metashape* (Agisoft LLC, 2021).

[45] QGIS Development Team. *QGIS Geographic Information System. Open Source Geospatial Foundation Project.* (2022).

[46] Yong Technology Inc. *GeoRose* (2014).

