# Supplementary material for: Eolian erosion of polygons in the Atacama Desert as a proxy for hyper-arid environments on Earth and beyond
Source: Sci Rep. 2022 Jul 20;12:12394. doi: 10.1038/s41598-022-16404-y (PMC9300690; doi:10.1038/s41598-022-16404-y)
Supplement: Supplementary file 1 — Supplementary Information 1. [file 41598_2022_16404_MOESM1_ESM.docx]

Supplementary file 1 - Appendix

Title: Eolian erosion of polygons in the Atacama Desert as a proxy for hyper-arid environments on Earth and beyond

Author list:

Christof Sager^1*^(christof.sager@tu-berlin.de), Alessandro Airo^1,2^ (alessandro.airo@mfn.berlin), Felix L. Arens^1^ (f.arens@tu-berlin.de), Dirk Schulze-Makuch^1,3,4,5^ (schulze-makuch@tu-berlin.de)

*^1^ Astrobiology Research Group, Zentrum für Astronomie und Astrophysik, Technische Universität Berlin,* *10623 Berlin, Germany*

*^2^ Museum für Naturkunde, Leibniz-Institut für Evolutions- und Biodiversitätsforschung, 10115 Berlin, Germany*

*^3^ Section Geomicrobiology, German Research Centre for Geosciences (GFZ), 14473 Potsdam, Germany*

*^4^ Department of Experimental Limnology, Leibniz-Institute of Freshwater Ecology and Inland Fisheries (IGB), 12587 Stechlin, Germany*

*^5^ School of the Environment, Washington State University, Pullman, WA 99164, USA*

*** Corresponding Author

Appendix 1. Detailed methods

Field work

Field work, preparations for photogrammetric reconstructions, and drone flight were conducted from 24.7.2019 to 19.8.2019 for the here studied polygonal network (PN; ‑24.07796, ‑69.99339) in the Yungay valley according to the methods used in Sager et al. 2021 [25]. The drone flight for the surrounding terrain was conducted in January 2017. Soil samples were collected within the three different polygon type areas (P1, P2, P3) along three separate soil transects stretching across a single polygon and its neighboring polygon shoulders. A total of 12 samples at P1, 11 samples at P2, and 12 samples P3, each ~250 g, were collected with a spoon and a hammer within a ~15 ×15 cm area down to 10 cm depth. At P3 a ~3 m long and maximal 60 cm deep trench was dug using a shovel and jackhammer. Within this trench, samples (~250 g each) were taken at two depth profiles: along a sand wedge (at 10, 20, 30, 45, 50 cm depth) and along the polygon, between the polygon center and the margin of the polygon (at 10, 20, 30, 40, 50, 60 cm depth).
In preparation for the drone flights and the photogrammetric reconstruction, 12 ground control points resulting in 11 scale bars were laid out in the study site. Additionally, we used six 100 cm long plastic tubes as check scale bars validating the accuracy of the photogrammetric reconstruction, as their lengths vary between 99.8 and 101.3 cm in the orthophoto (Supplementary File 3). The distances between the markers and the relative altitudes were measured using a laser rangefinder. The flight mission for the PN was carried out using primarily parallel flight paths at ~7 m flying altitude with a downward oriented camera (pitch 0°) of DJI Phantom 4 and 80% front and 40% side image overlap.

Photogrammetric reconstruction

The image data sets was processed in “AgiSoft Metashape” to generate 3D spatial data, digital elevation models (DEM) and orthophotos for here studied PN [42]. An overview model that displays parts of the alluvial fan on which the polygonal network is located, and a close-up model of the polygonal network were generated. The processing report of the respective AgiSoft projects, including important parameters (number of photos, resolutions of orthophotos and DEMs) and errors (e.g., reprojection error) are available at Supplementary File 3. To vertically scale the 3D dataset of the close-up model, we used the height differences between ground control points that were measured in the field. They were converted to absolute heights by defining the software-based elevation estimation (based on drone GPS data of the images) of one ground control point as correct and subtracting or adding the relative elevation to the one ground control point.

GIS analyses

Polygons completely enclosed by clearly visible sand wedge or outlined by troughs were mapped and analyzed in the software QGIS using the generated orthophoto and DEM of the PN [43]. The polygonal networks visible in the overview model were mapped similar, but the polygons were not mapped individually but in clusters. Intersection points were generated where three or more polygons touch. In those cases, where a boulder covers the intersection point or parts of the polygon geometry, intersection position and polygon mapping were estimated manually. Polygon orientation is based on the orientation of the long side of a minimum oriented bounding rectangle and Sand wedge orientation is based on the line orientation between intersections. Their orientation can be described by e.g., 90° (east) or by 270° (west), representing the same orientation. Therefore, rose diagrams generated with the software GeoRose are mirrored, while the direction of the slope is unidirectional [44]. To calculate the through wall slopes and polygon shoulder elevations a 10 cm buffer was created along each side of the sand wedge centerline (Supplementary Figure S 4), while the area around the intersection points was excluded (circle with 20 cm radius). The channel network was calculated based on the DEM of the overview model by using the Strahler Order algorithm in QGIS. The DEM was preprocessed, by interpolating no data values and applying a fill sinks algorithm to provide outflows from the DEM. Further statistical parameters were calculated in Microsoft Excel or using a script written in the scripting language Python, e.g., slope direction for each polygon (Supplementary File 2). For the calculation of the intersection angle, polygons located at the margin of the mapped area were excluded, since a neighboring polygon with an intersecting sand wedge is absent.

Laboratory analyses

For the determination of the mineralogical composition of the samples, powder X-Ray Diffraction (XRD) analysis including a semi-quantitative XRD (SQ-XRD) analysis was performed by the authors on all soil samples using a Bruker D2 Phaser benchtop diffractometer with Cu radiation and a Ni filter at the Department of Applied Geochemistry (Technische Universität Berlin).

In preparation for XRD analysis, the soil samples were pre-pulverized by mortar and pestle (10 min) and then dry-grinded for 20 minutes in the XRD-Mill McCrone (~3 ml sample volume). For XRD analysis, samples were scanned between 3° (2*θ* = *2Theta*) and 80° (2*θ*) at 30 kV and 10 mA using a step size of 0.1° (2*θ*) and a count time of 2 s. Scan time was about 70 min with a 1 mm divergence slit, and 1 mm air scattering screen. A LYNXEYE detector (opening 4° 2*θ*) was used and the lower and upper discriminator was set to 0.180 V and 0.280 V. SQ-XRD analysis was performed to determine the mineral content (in percent) based on the peak intensities of the mineral assemblage using the “Diffrac.eva” software and the “Powder Diffraction File Minerals 2019” (database of the *international centre for diffraction data*).

The salt content by weight (wt%) was determined by measuring the weight loss of pulverized samples (~5 g) after leaching in 4 - 6 l de-ionized water. After the material settled from suspension the salt solution was decanted and centrifuged. Possible sediment residue was returned to the material and refilled with de-ionized water. These steps were repeated until the electronic conductivity of the leachate solution remained constant below 100 μS. The leached samples were dried at 50 °C and weighed. To evaluate the reproducibility of the salt content, three sub-samples of the polygon depth profile at P3 in 10 cm depth were analyzed showing salt contents of 23, 21, and 22 wt%. The absolute salt content varies between results from the sample leaching method and the determination via SQ-XRD. Especially for samples containing sulfates discrepancies are larger, resulting in lower salt contents via SQ-XRD compared to sample leaching. However, as the trends between those methods remain similar, comparability between the samples is retained (Supplementary Fig. S 2). The grain size distribution analysis is based on weighed grain size fractions, therefore, ~60-90 g sample material was leached in de-ionized water until a constant electrical conductivity below 100 μS was measured. The samples were then wet sieved using de-ionized water and a mechanical shaker with mesh sizes: 4000 μm, 2000 μm, 600 μm, 200 μm, and 63 μm. The fraction <63 μm was centrifuged in a Sigma 3-16 centrifuge to separate the silt-sized from the clay-sized fraction.

Appendix 2. Supplemental Figures


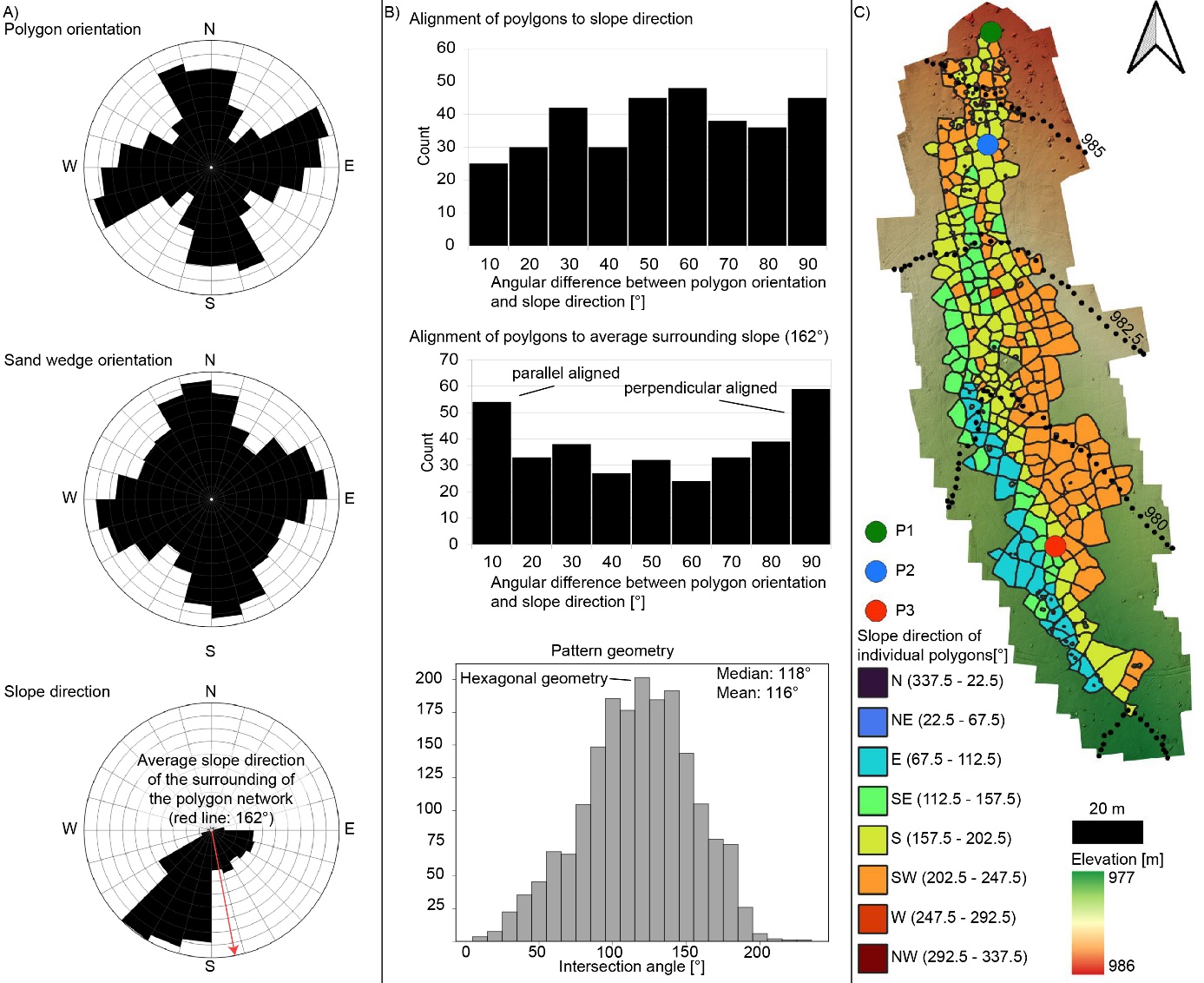


**Figure S 1.** Orientational and geometrical data for the polygonal network. (A) Rose diagrams for polygon orientation, sand wedge orientation and slope direction of the polygons. Note the red line indicating the average slope for the surrounding area (bounding box from Fig. 1). The height of the black bars represents relative abundance of the respective orientation or direction. (B) From top to bottom: histograms showing the alignment of the polygons to the individual slope direction, the alignment of polygons to the average surrounding slope, and the pattern geometry based on the abundance of intersection angles. Note that the polygons are roughly aligned with the average slope of 182°, but not with the individual slope based on the close-up DEM. (D) Histogram of intersection angles. As 120° intersection angles are most abundant, the polygonal network geometry is primarily hexagonal. (C) Mapped polygons are color-coded based on their slope direction, corresponding to the rose diagram of the slope direction. Elevation is given in meter above sea level.


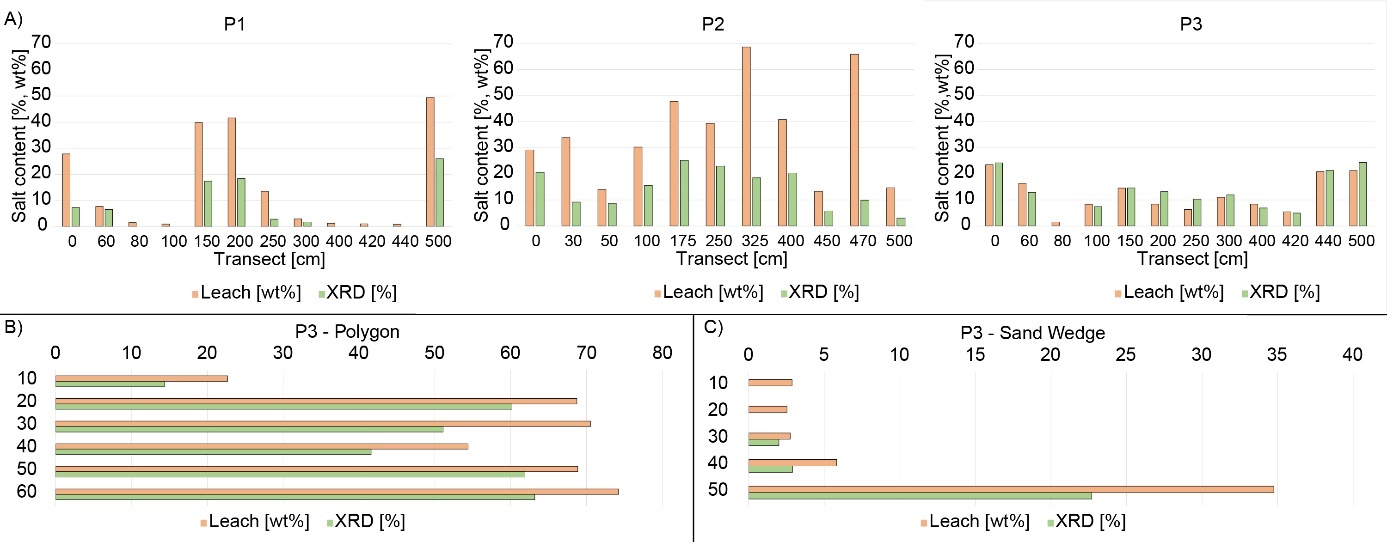


**Figure S 2.** Comparison of the salt content determined by leaching with de-ionized water (light orange bars) and from semi-quantitative X-Ray diffraction (XRD, light green bars) for (A) P1, P2, P3 and the depth profiles at P3 for (B) the polygon and (C) the sand wedge. Note that the salt content by leaching shows a similar trend as salt content from XRD, but a general higher salt content for samples of P1 and P2, which do not contain highly soluble salts (see Fig. 2).


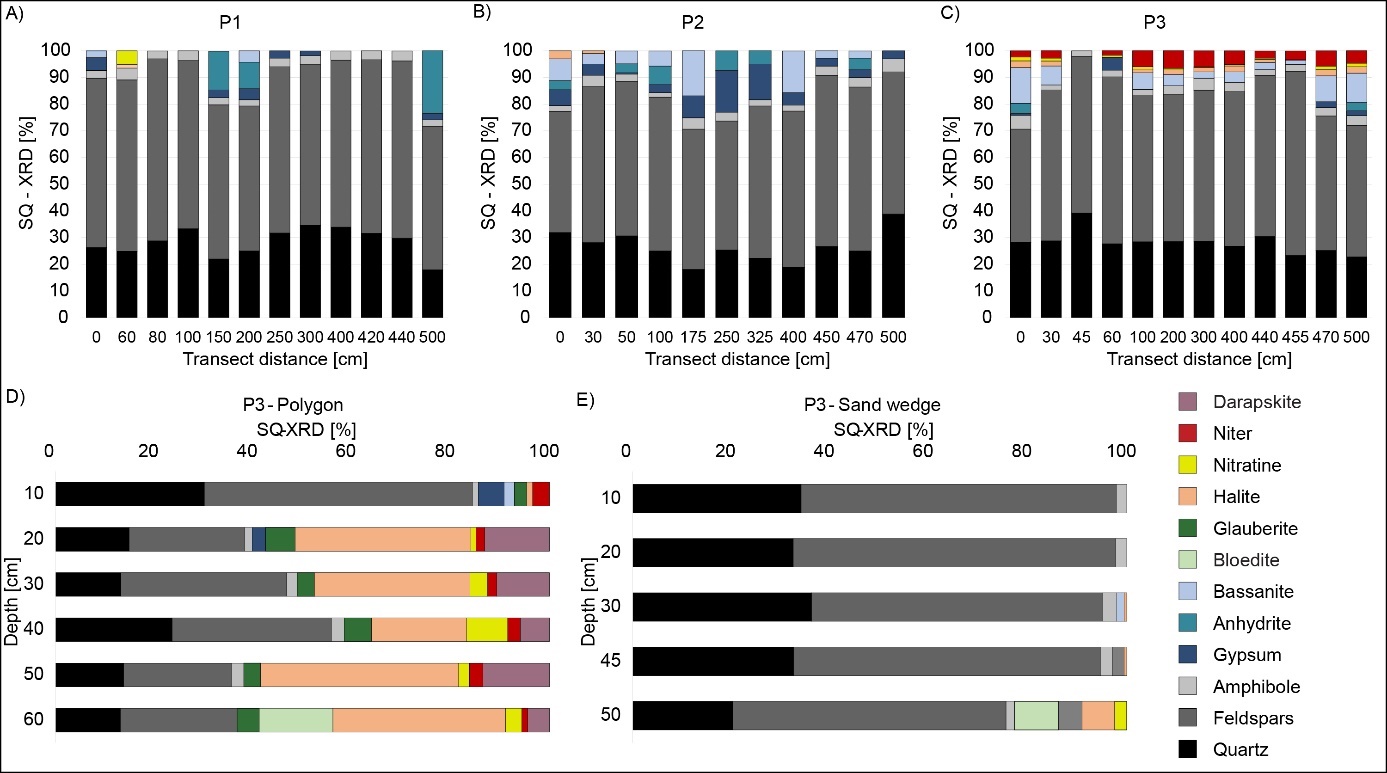


**Figure S 3.** Semi-quantitative X-Ray diffraction (SQ-XRD) results showing the mineralogical composition along (A, B, C) the soil transects and, depth profiles of (D) the polygon and (E) the sand wedge at P3.


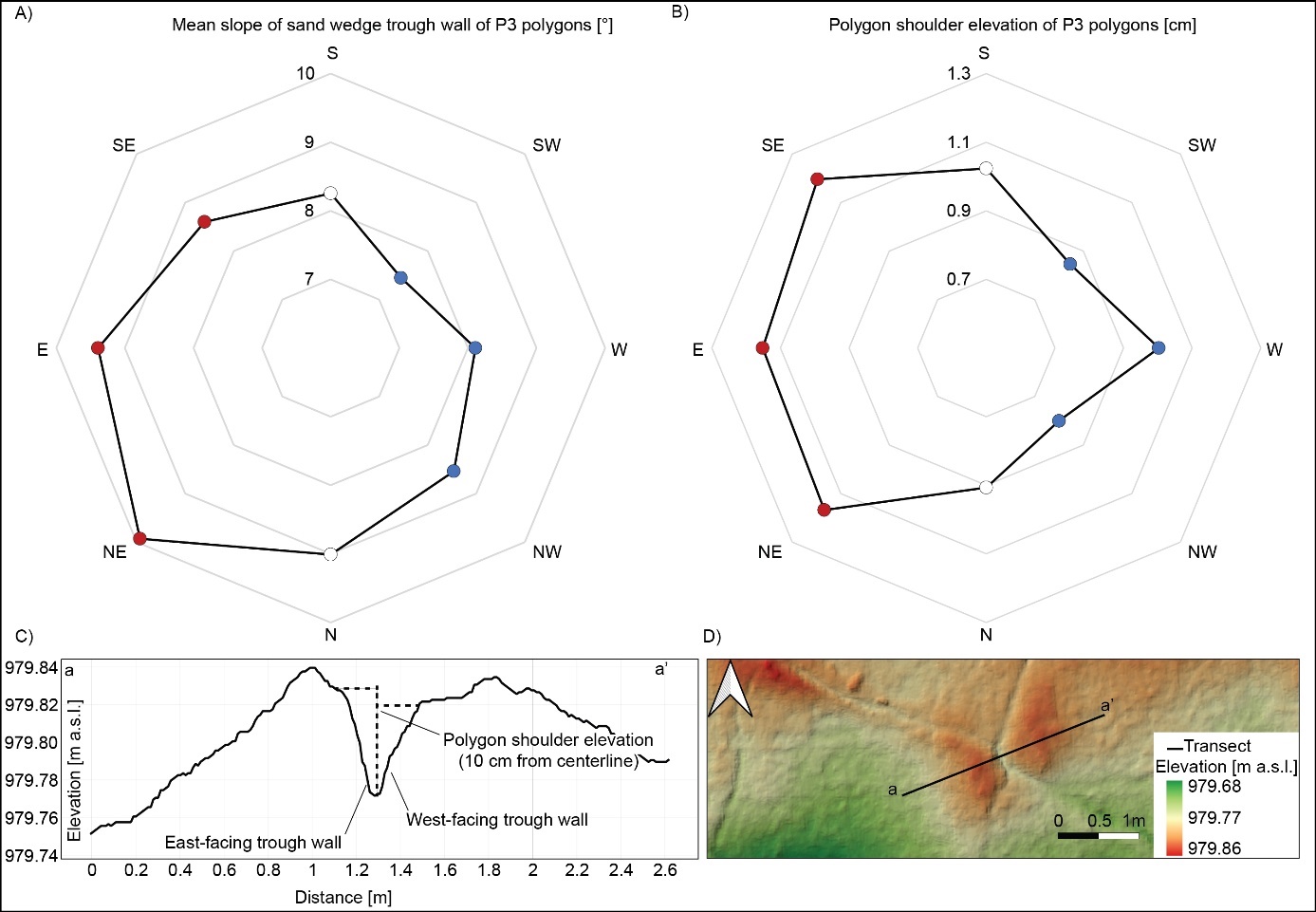


**Figure S 4**. Results of the morphological trough analysis of sand wedges. (A) Diagram showing the mean slope of the sand wedge trough walls and their respective slope direction. Red dots represent wind sheltered directions (leeward); blue dots represent wind facing direction and white dots represent directions perpendicular to the main wind direction, being from west to east. Note that e.g., S represents south-facing trough walls and SW southwest-facing trough walls. (B) Diagram showing the polygon shoulder elevation of the respective slope direction categories, e.g., east-facing (red) polygon shoulders show 1.15 cm elevation, while west-facing (blue) polygon shoulders show 1.00 cm elevation. (C) Schematic example of a trough showing two trough walls and that the polygon shoulder elevation is calculated 10 cm from the mapped centerline. (D) DEM corresponding to the transect (black line) from (C).


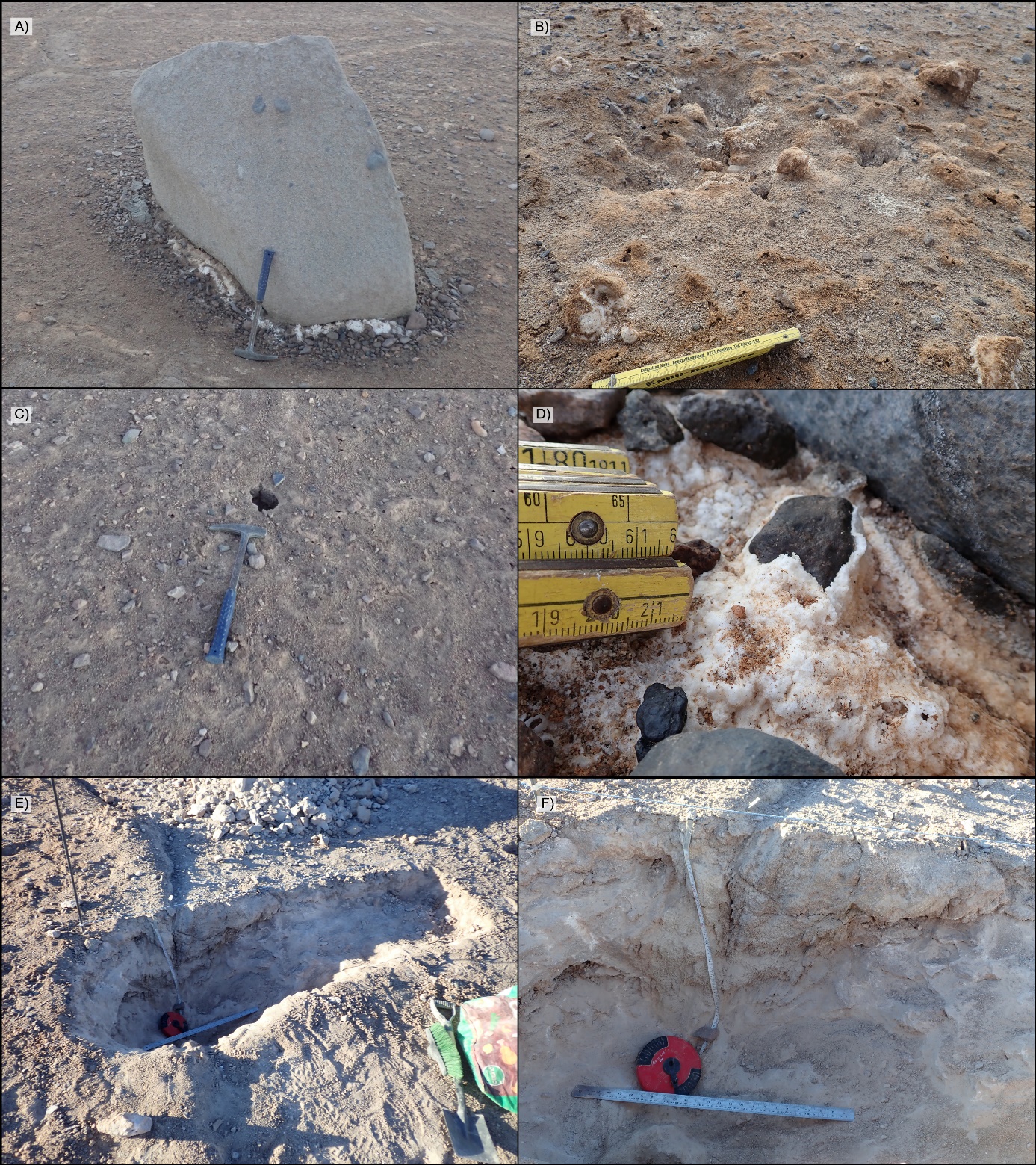


**Figure S 5.** Field photographs showing (A) a ~80 cm tall boulder sitting on elevated salt crusts located on a P3 type polygon, (B) moist, dark brown patches of salt crusts in the morning at P3 surfaces, (C) surficial cavities, and (D) salt encrusted clasts. (E) The excavated soil pit at P3. The metal ruler is 60 cm long. (F) Close-up of the soil pit at P3 with the ~50 cm deep sand wedge located right of the measuring tape.
