# Supplementary material for: Eolian erosion of polygons in the Atacama Desert as a proxy for hyper-arid environments on Earth and beyond
Source: Sci Rep. 2022 Jul 20;12:12394. doi: 10.1038/s41598-022-16404-y (PMC9300690; doi:10.1038/s41598-022-16404-y)
Supplement: Supplementary file 3 — Supplementary Information 3. [file 41598_2022_16404_MOESM3_ESM.pdf]

Processing Report

Close-up model

14 February 2022

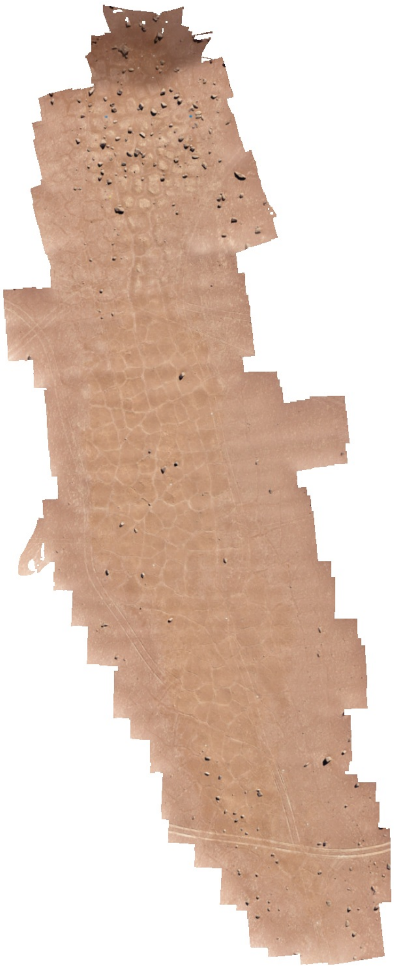

Survey Data

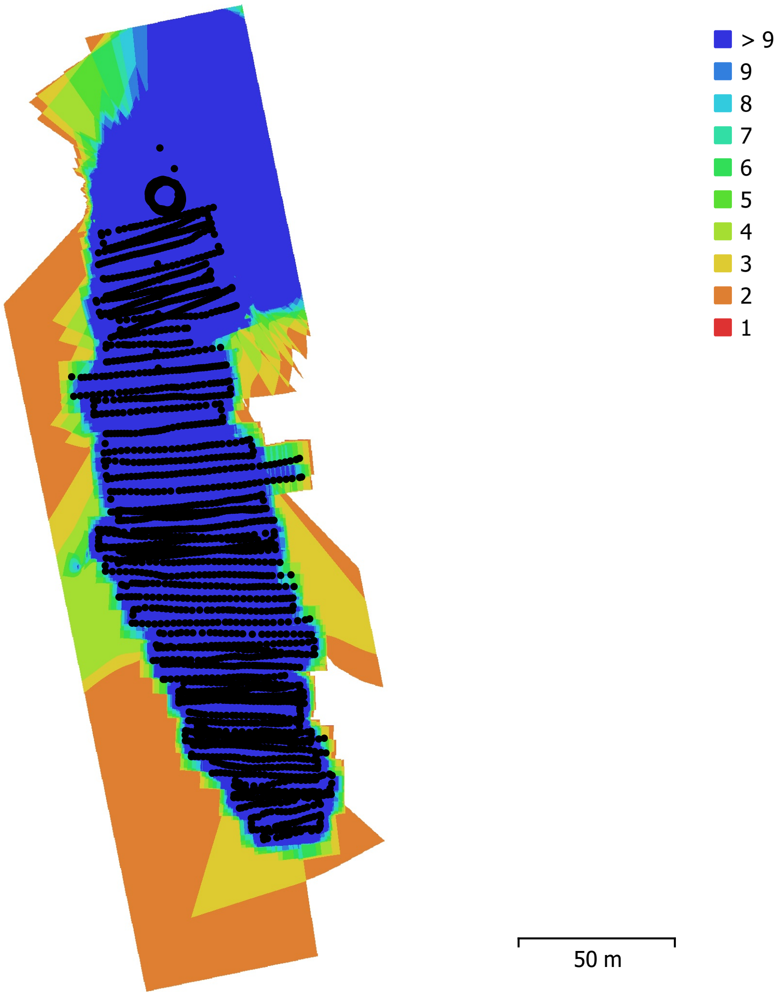

Fig. 1. Camera locations and image overlap.

Number of images:	2,609	Camera stations:	2,410
Flying altitude:	6.35 m	Tie points:	1,246,878
Ground resolution:	2.62 mm/pix	Projections:	8,417,258
Coverage area:	0.0235 km²	Reprojection error:	2.02 pix

**Camera Model**	**Resolution**	**Focal Length**	**Pixel Size**	**Precalibrated**
FC330 (3.61mm)	4000 x 3000	3.61 mm	1.56 x 1.56 μm	No

Table 1. Cameras.

Camera Calibration

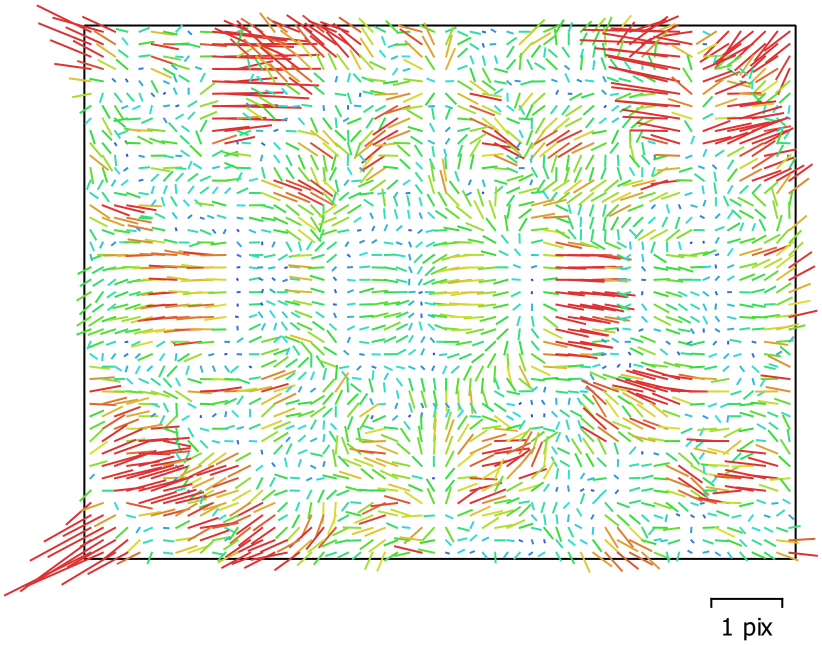

Fig. 2. Image residuals for FC330 (3.61mm).

FC330 (3.61mm)

2586 images

Type	Resolution	Focal Length	Pixel Size
**Frame**	**4000 x 3000**	**3.61 mm**	**1.56 x 1.56 µm**

	**Value**	**Error**	**F**	**Cx**	**Cy**	**B1**	**B2**	**K1**	**K2**	**K3**	**K4**	**P1**	**P2**
**F**	**2339**	0.14	1.00	0.05	-0.77	-0.07	-0.08	-0.05	0.05	-0.05	0.05	0.01	-0.02
**Cx**	**-93.0018**	0.057		1.00	-0.05	-0.00	0.74	-0.00	0.01	-0.01	0.01	0.40	0.05
**Cy**	**26.444**	0.15			1.00	-0.49	0.07	-0.02	0.01	-0.01	0.00	-0.01	0.14
**B1**	**-2.04408**	0.039				1.00	-0.01	0.02	-0.01	0.01	-0.01	0.01	-0.02
**B2**	**-19.5324**	0.039					1.00	0.00	0.01	-0.00	0.00	0.03	0.16
**K1**	**-0.000741133**	4e-05						1.00	-0.95	0.91	-0.86	-0.02	-0.03
**K2**	**-0.00234595**	0.00013							1.00	-0.99	0.96	0.00	0.00
**K3**	**0.000906816**	0.00017								1.00	-0.99	-0.00	-0.00
**K4**	**0.000644588**	7.5e-05									1.00	0.00	0.00
**P1**	**-0.000695161**	3.4e-06										1.00	-0.03
**P2**	**-7.23814e-05**	4.2e-06											1.00

Table 2. Calibration coefficients and correlation matrix.

Camera Locations

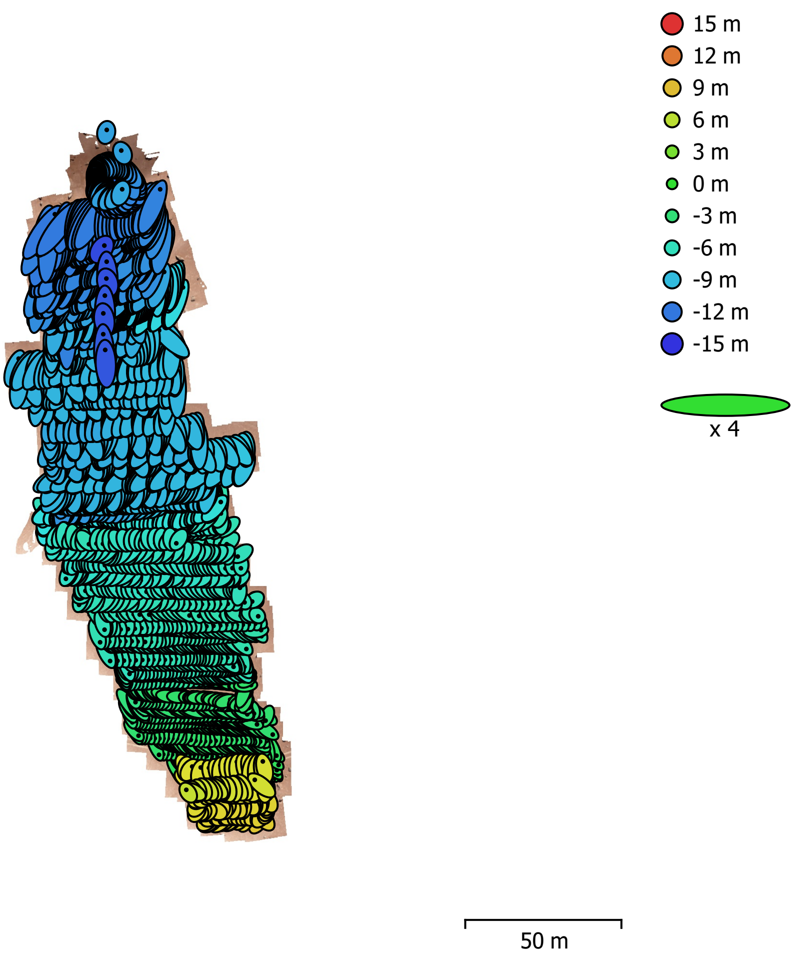

Fig. 3. Camera locations and error estimates.
Z error is represented by ellipse color. X,Y errors are represented by ellipse shape.
Estimated camera locations are marked with a black dot.

X error (m)	Y error (m)	Z error (m)	XY error (m)	Total error (m)
0.637507	1.43106	8.02782	1.56664	8.17926

Table 3. Average camera location error.
X - Longitude, Y - Latitude, Z - Altitude.

Ground Control Points

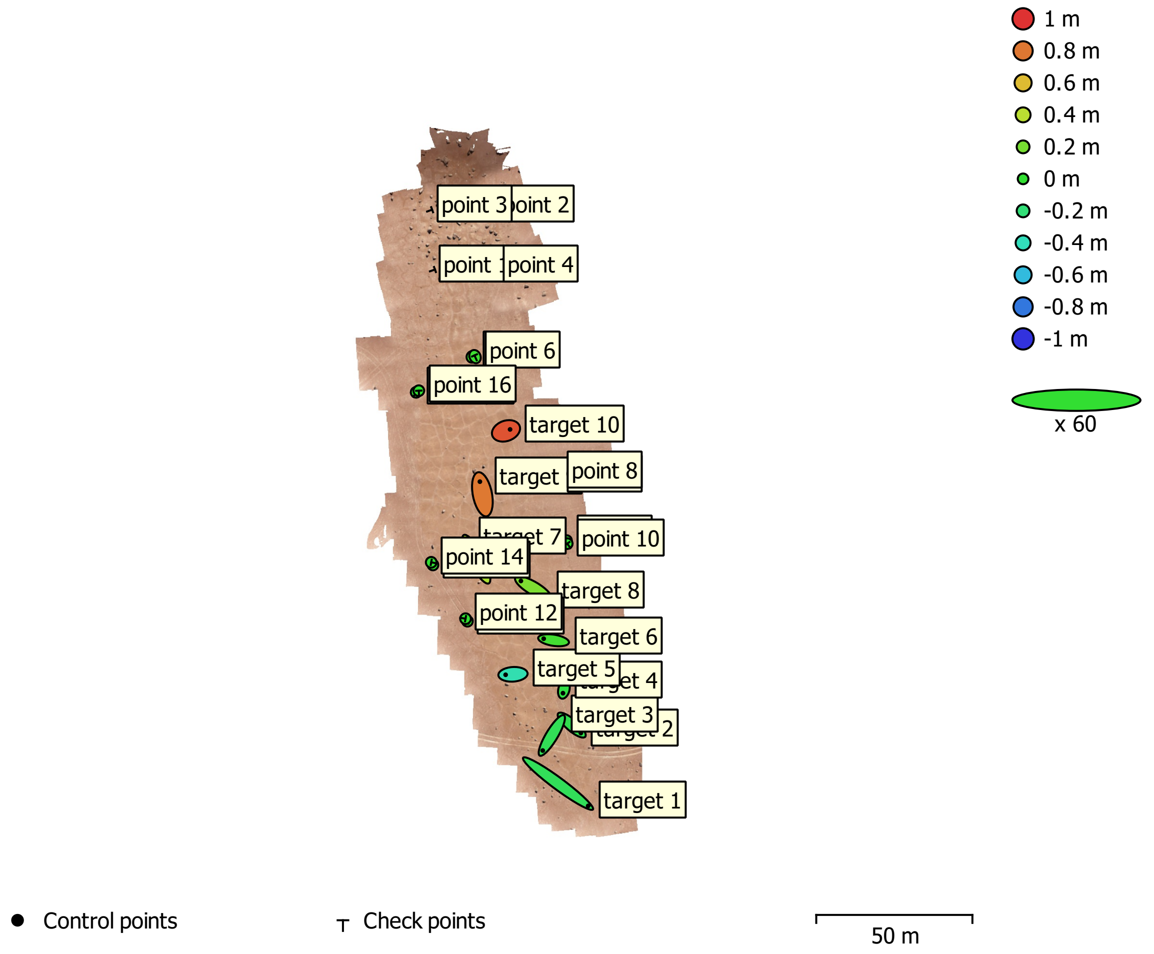

Fig. 4. GCP locations and error estimates.
Z error is represented by ellipse color. X,Y errors are represented by ellipse shape.
Estimated GCP locations are marked with a dot or crossing.

Count	X error (cm)	Y error (cm)	Z error (cm)	XY error (cm)	Total (cm)
10	13.0209	12.3386	42.3563	17.9383	45.9983

Table 4. Control points RMSE.
X - Longitude, Y - Latitude, Z - Altitude.

Count	X error (cm)	Y error (cm)	Z error (cm)	XY error (cm)	Total (cm)
12	0.165491	0.384025	1.10568	0.418165	1.18211

Table 5. Check points RMSE.
X - Longitude, Y - Latitude, Z - Altitude.

Label	X error (cm)	Y error (cm)	Z error (cm)	Total (cm)	Image (pix)
target 1	32.1665	-23.8219	-11.3435	41.6034	1.337 (18)
target 2	9.7698	-8.45516	-8.09788	15.2484	1.450 (21)
target 3	-9.70823	-16.1299	-10.5216	21.5668	1.426 (29)
target 4	-0.9852	-3.90475	-4.77332	6.24517	1.559 (15)
target 5	-7.83171	-0.494448	-36.7435	37.5721	1.529 (32)
target 6	-10.5318	1.62632	4.87313	11.718	1.448 (35)
target 7	-9.82917	18.987	35.0797	41.0817	1.376 (28)
target 8	-13.2343	7.77847	21.9774	26.8078	0.685 (10)
target 9	-2.66299	13.4788	79.4539	80.633	1.530 (21)
target 10	4.35571	1.55917	90.6228	90.7408	1.616 (23)
**Total**	**13.0209**	**12.3386**	**42.3563**	**45.9983**	**1.449**

Table 6. Control points.
X - Longitude, Y - Latitude, Z - Altitude.

Label	X error (cm)	Y error (cm)	Z error (cm)	Total (cm)	Image (pix)
point 1					1.324 (27)
point 2					1.785 (20)
point 3					2.437 (15)
point 4					1.387 (27)
point 5	-0.067731	-0.329152	2.32882	2.35294	2.920 (31)
point 6	-0.569262	1.28894	3.04086	3.35146	1.630 (27)
point 7	-2.21158e-06	-2.83272e-06	1.66721e-07	3.59767e-06	0.000 (19)
point 8	5.00286e-07	-2.96798e-07	-2.4904e-06	2.55743e-06	0.000 (19)
point 9	3.44955e-06	-4.84742e-06	1.47922e-06	6.13066e-06	0.000 (13)
point 10	-5.89979e-06	8.17165e-06	1.05186e-06	1.01336e-05	0.000 (12)
point 11	-4.34666e-06	-1.78e-06	-3.86913e-06	6.08539e-06	0.000 (24)
point 12	2.0599e-06	-3.6288e-07	2.61204e-06	3.34629e-06	0.000 (23)
point 13	1.79895e-06	-6.26368e-06	1.15498e-05	1.32615e-05	0.000 (15)
point 14	-5.2006e-06	9.82378e-07	-1.42374e-05	1.51893e-05	0.000 (15)
point 15	5.65783e-06	9.00452e-06	1.59277e-05	1.91516e-05	0.000 (22)
point 16	-1.33621e-07	2.93746e-06	-3.8888e-07	2.9661e-06	0.000 (16)

Label	X error (cm)	Y error (cm)	Z error (cm)	Total (cm)	Image (pix)
Total	0.165491	0.384025	1.10568	1.18211	1.193

Table 7. Check points.

X - Longitude, Y - Latitude, Z - Altitude.

Scale Bars

Label	Distance (m)	Error (m)
target 1_target 2	23.4709	0.230942
target 1_target 3	22.945	0.195003
target 2_target 4	13.9882	0.0381709
target 4_target 6	18.5049	0.0648676
target 5_target 6	16.7908	-2.27922
target 6_target 8	19.8887	0.138693
target 7_target 8	21.3876	0.077609
target 7_target 9	19.5841	0.144055
target 9_target 10	19.3757	0.225671
point 1_point 4	19.9962	-0.0038459
point 2_point 3	20.0038	0.00375571
**Total**		**0.699925**

Table 8. Control scale bars.

Label	Distance (m)	Error (m)
point 5_point 6	1.00663	
point 7_point 8	0.998449	
point 9_point 10	1.01197	
point 11_point 12	1.01212	
point 13_point 14	1.01291	
point 15_point 16	1.00744	
**Total**		

Table 9. Check scale bars.

Digital Elevation Model

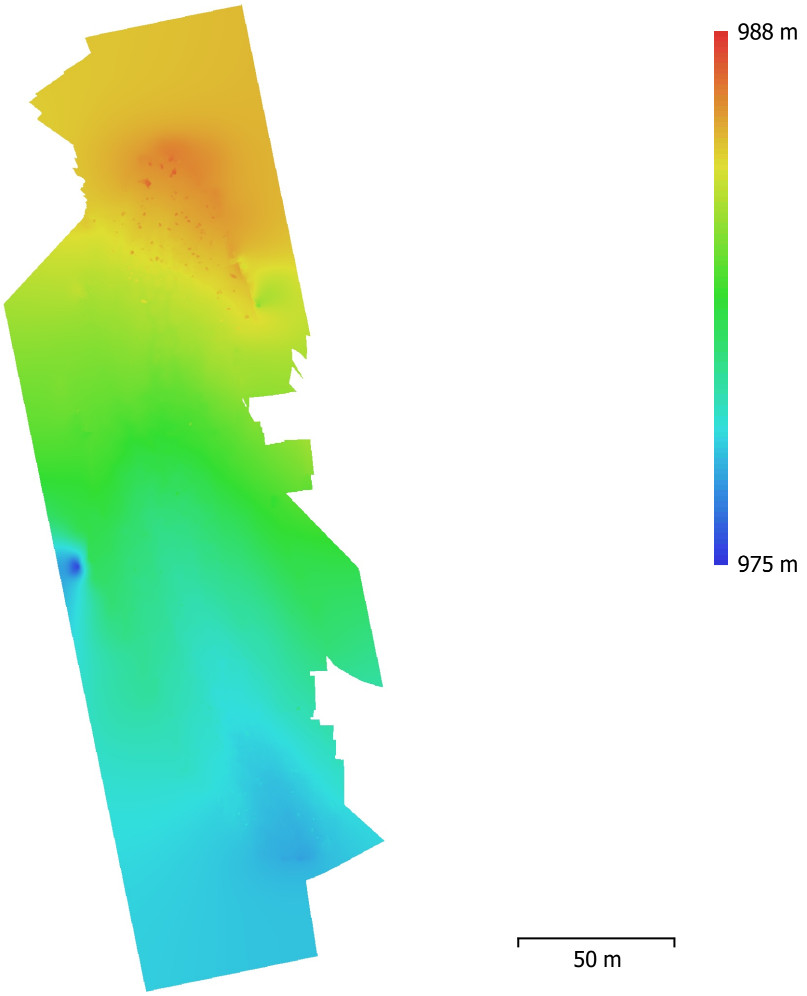

Fig. 5. Reconstructed digital elevation model.

Resolution:	1.05 cm/pix
Point density:	0.909 points/cm²

Processing Parameters

**General**	
Cameras	2586
Aligned cameras	2410
Markers	26
Scale bars	17
Coordinate system	WGS 84 (EPSG::4326)
Rotation angles	Yaw, Pitch, Roll
**Point Cloud**	
Points	1,246,878 of 1,400,082
RMS reprojection error	0.280094 (2.02253 pix)
Max reprojection error	1.27106 (107.56 pix)
Mean key point size	7.47448 pix
Point colors	3 bands, uint8
Key points	No
Average tie point multiplicity	7.38743
**Alignment parameters**	
Accuracy	Medium
Generic preselection	Yes
Reference preselection	Source
Key point limit	40,000
Tie point limit	4,000
Filter points by mask	Yes
Mask tie points	No
Guided image matching	No
Adaptive camera model fitting	Yes
Matching time	14 minutes 32 seconds
Matching memory usage	986.60 MB
Alignment time	44 minutes 9 seconds
Alignment memory usage	604.41 MB
**Optimization parameters**	
Parameters	f, b1, b2, cx, cy, k1-k4, p1, p2
Adaptive camera model fitting	Yes
Optimization time	1 minutes 0 seconds
Date created	2020:12:04 10:02:51
Software version	1.6.5.11105
File size	190.68 MB
**Depth Maps**	
Count	2410
**Depth maps generation parameters**	
Quality	Medium
Filtering mode	Mild
Processing time	2 hours 14 minutes
Memory usage	1.17 GB
Date created	2020:12:04 14:29:03
Software version	1.6.5.11105
File size	2.19 GB
**Dense Point Cloud**	
Points	109,052,800
Point colors	3 bands, uint8
**Depth maps generation parameters**	

Quality	Medium
Filtering mode	Mild
Processing time	2 hours 14 minutes
Memory usage	1.17 GB
**Dense cloud generation parameters**	
Processing time	1 hours 5 minutes
Memory usage	5.03 GB
Date created	2020:12:04 15:34:17
Software version	1.6.5.11105
File size	1.39 GB
**Tiled Model**	
Texture	3 bands, uint8
**Depth maps generation parameters**	
Quality	Medium
Filtering mode	Mild
Processing time	2 hours 14 minutes
Memory usage	1.17 GB
**Reconstruction parameters**	
Source data	Dense cloud
Tile size	512
Face count	Medium
Enable ghosting filter	Yes
Processing time	3 hours 16 minutes
Memory usage	2.30 GB
Date created	2021:01:25 12:55:13
Software version	1.7.1.11797
File size	2.42 GB
**DEM**	
Size	14,661 x 30,123
Coordinate system	WGS 84 (EPSG::4326)
**Reconstruction parameters**	
Source data	Dense cloud
Interpolation	Enabled
Processing time	8 minutes 1 seconds
Memory usage	370.31 MB
Date created	2020:12:07 09:48:11
Software version	1.6.5.11105
File size	559.43 MB
**Orthomosaic**	
Size	46,583 x 120,491
Coordinate system	WGS 84 (EPSG::4326)
Colors	3 bands, uint8
**Reconstruction parameters**	
Blending mode	Mosaic
Surface	DEM
Enable hole filling	Yes
Processing time	42 minutes 42 seconds
Memory usage	3.39 GB
Date created	2020:12:04 16:18:16
Software version	1.6.5.11105
File size	37.52 GB
**System**	
Software name	Agisoft Metashape Professional
Software version	1.8.0 build 13794
OS	Windows 64 bit
RAM	15.93 GB

CPU	AMD Ryzen 5 3600 6-Core Processor
GPU(s)	NVIDIA GeForce GTX 1660 SUPER

Processing Report

Overview model

14 February 2022

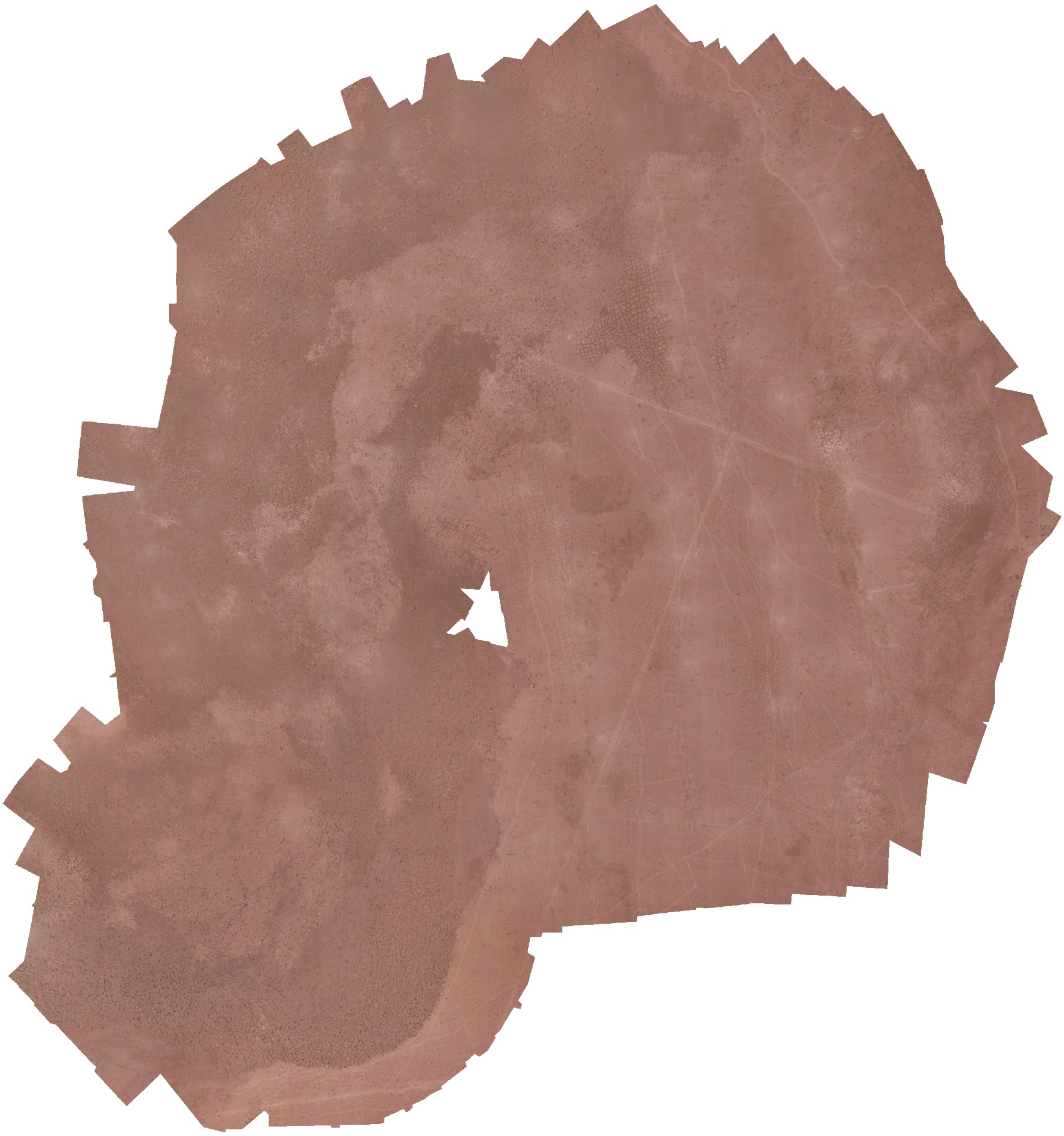

Survey Data

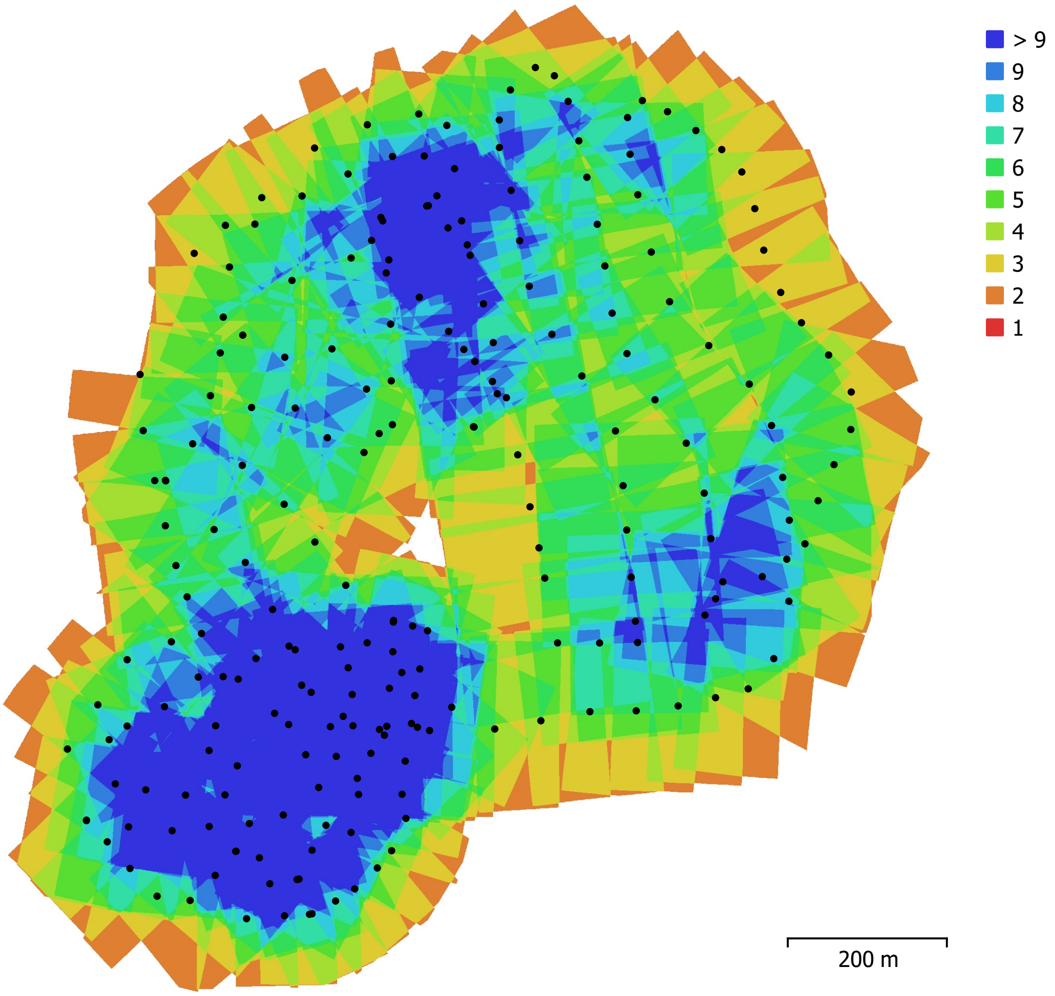

Fig. 1. Camera locations and image overlap.

Number of images:	295	Camera stations:	228
Flying altitude:	117 m	Tie points:	205,110
Ground resolution:	4.65 cm/pix	Projections:	604,516
Coverage area:	0.966 km²	Reprojection error:	0.726 pix

Camera Model	Resolution	Focal Length	Pixel Size	Precalibrated
FC300C (3.61 mm)	4000 x 3000	3.61 mm	1.56 x 1.56 μm	No

Table 1. Cameras.

Camera Calibration

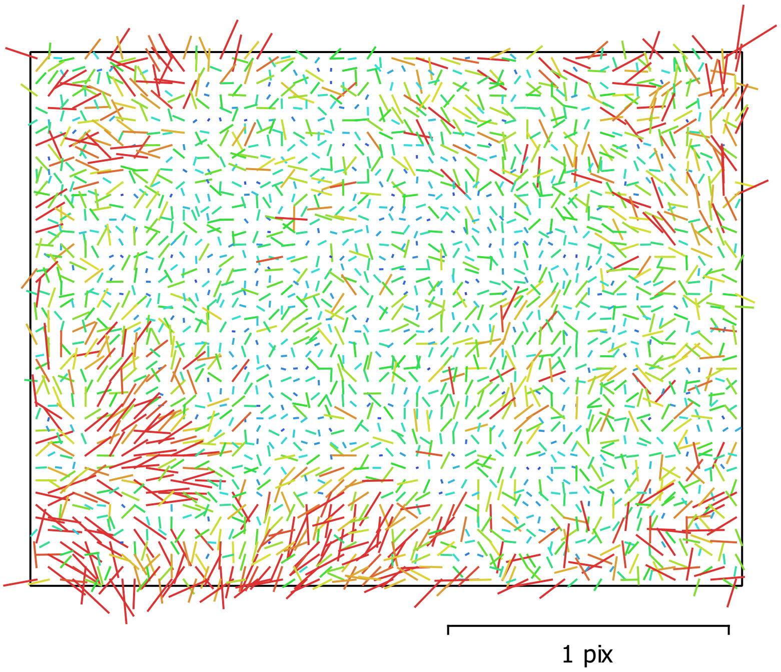

Fig. 2. Image residuals for FC300C (3.61 mm).

FC300C (3.61 mm)

295 images

Type	Resolution	Focal Length	Pixel Size
**Frame**	**4000 x 3000**	**3.61 mm**	**1.56 x 1.56 µm**

	Value	Error	Cx	Cy	B1	B2	K1	K2	K3	K4	P1	P2
**F**	**2311.25**											
**Cx**	**-0.187136**	0.08	1.00	-0.08	-0.11	-0.07	-0.05	0.02	-0.03	0.03	-0.24	0.01
**Cy**	**19.7774**	0.078		1.00	0.06	-0.14	0.00	-0.01	0.01	-0.02	0.03	-0.41
**B1**	**-3.95601**	0.0068			1.00	-0.01	0.04	-0.03	0.03	-0.02	0.10	-0.07
**B2**	**-0.102074**	0.0067				1.00	0.00	-0.00	0.00	-0.00	0.05	0.12
**K1**	**-0.104378**	9.3e-05					1.00	-0.83	0.79	-0.73	0.12	0.01
**K2**	**0.0853783**	0.00025						1.00	-0.99	0.96	-0.01	0.00
**K3**	**-0.0357316**	0.00032							1.00	-0.99	0.01	-0.01
**K4**	**0.0104403**	0.00014								1.00	-0.01	0.01
**P1**	**0.000453224**	3.1e-06									1.00	0.04
**P2**	**-0.00014224**	2.9e-06										1.00

Table 2. Calibration coefficients and correlation matrix.

Camera Locations

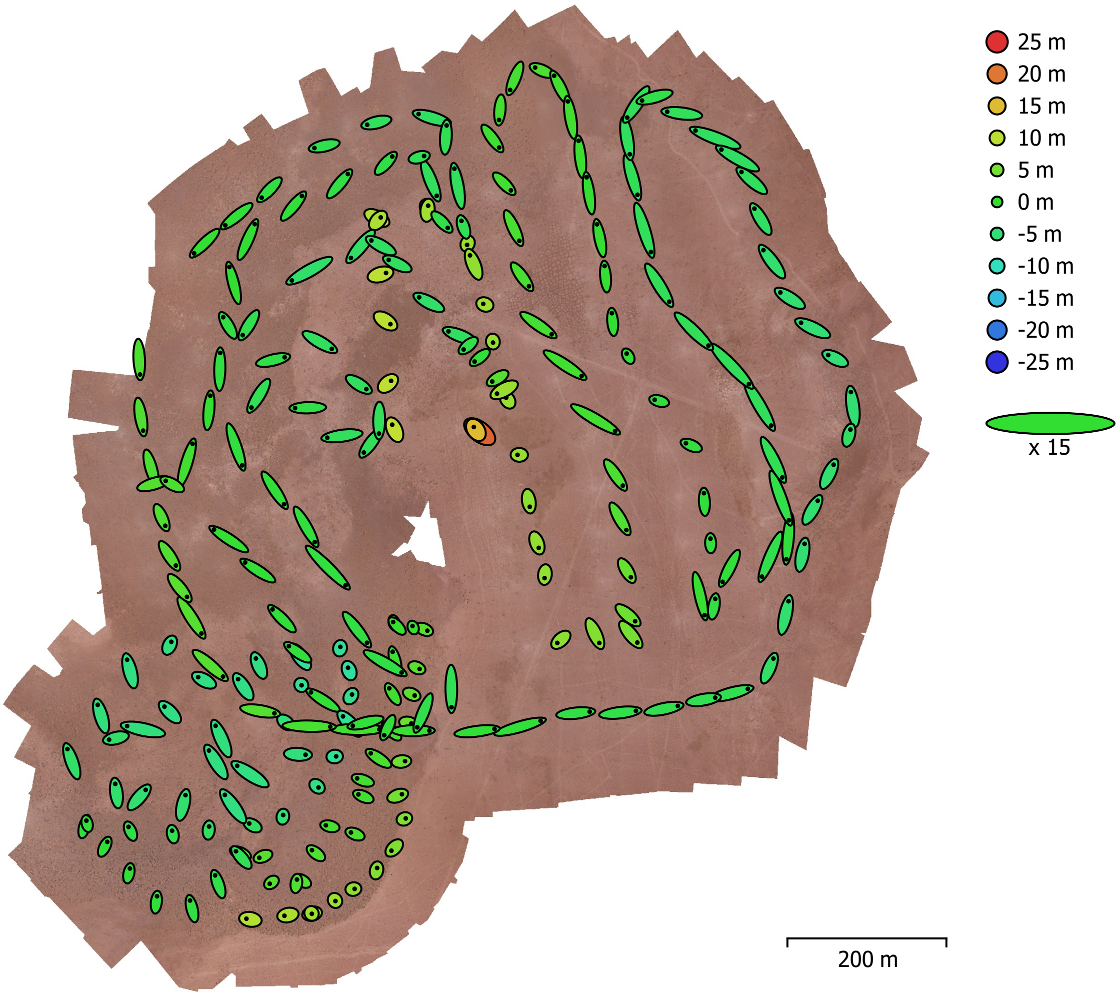

Fig. 3. Camera locations and error estimates.
Z error is represented by ellipse color. X,Y errors are represented by ellipse shape.
Estimated camera locations are marked with a black dot.

X error (m)	Y error (m)	Z error (m)	XY error (m)	Total error (m)
1.31753	1.49932	4.38904	1.99596	4.82157

Table 3. Average camera location error.
X - Longitude, Y - Latitude, Z - Altitude.

Digital Elevation Model

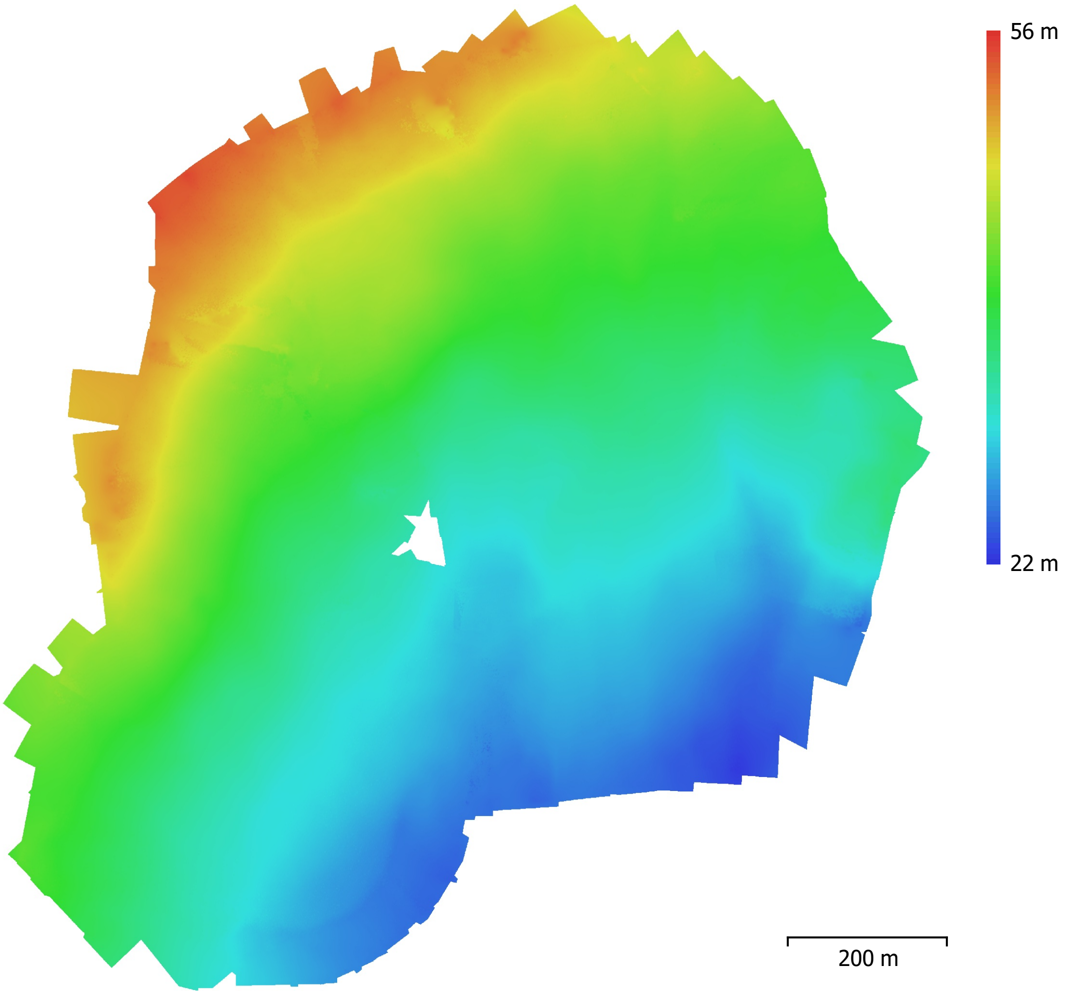

Fig. 4. Reconstructed digital elevation model.

Resolution:	9.3 cm/pix
Point density:	116 points/m²

Processing Parameters

**General**	
Cameras	295
Aligned cameras	228
**Shapes**	
Point	2
LineString	1181
Polygon	60491
Coordinate system	WGS 84 (EPSG::4326)
Rotation angles	Yaw, Pitch, Roll
**Point Cloud**	
Points	205,110 of 276,515
RMS reprojection error	0.391346 (0.725937 pix)
Max reprojection error	0.999989 (15.5816 pix)
Mean key point size	1.87199 pix
Point colors	3 bands, uint8
Key points	No
Average tie point multiplicity	3.33929
**Alignment parameters**	
Accuracy	Highest
Generic preselection	Yes
Reference preselection	Yes
Key point limit	40,000
Tie point limit	4,000
Adaptive camera model fitting	Yes
Matching time	6 minutes 6 seconds
Alignment time	3 minutes 25 seconds
**Optimization parameters**	
Parameters	b1, b2, cx, cy, k1-k4, p1, p2
Optimization time	7 seconds
File size	19.60 MB
**Depth Maps**	
Count	227
**Depth maps generation parameters**	
Quality	High
Filtering mode	Mild
Max neighbors	16
Processing time	8 minutes 59 seconds
Memory usage	2.70 GB
Date created	2022:01:03 14:09:47
Software version	1.8.0.13794
File size	1.05 GB
**Dense Point Cloud**	
Points	74,747,993
Point colors	3 bands, uint8
**Depth maps generation parameters**	
Quality	High
Filtering mode	Mild
Max neighbors	16
Processing time	8 minutes 59 seconds
Memory usage	2.70 GB

**Dense cloud generation parameters**	
Processing time	11 minutes 46 seconds
Memory usage	4.53 GB
Date created	2022:01:03 16:08:47
Software version	1.8.0.13794
File size	1.64 GB
**Model**	
Faces	9,917,790
Vertices	4,960,680
Vertex colors	3 bands, uint8
**Depth maps generation parameters**	
Quality	High
Filtering mode	Mild
Max neighbors	16
Processing time	8 minutes 59 seconds
Memory usage	2.70 GB
**Reconstruction parameters**	
Surface type	Arbitrary
Source data	Dense cloud
Interpolation	Enabled
Strict volumetric masks	No
Processing time	18 minutes 24 seconds
Memory usage	9.25 GB
Date created	2022:01:04 09:44:44
Software version	1.8.0.13794
File size	227.03 MB
**DEM**	
Size	20,587 x 22,586
Coordinate system	WGS 84 (EPSG::4326)
**Reconstruction parameters**	
Source data	Dense cloud
Interpolation	Enabled
Processing time	1 minutes 47 seconds
Memory usage	313.27 MB
Date created	2022:01:04 10:32:04
Software version	1.8.0.13794
File size	458.43 MB
**Orthomosaic**	
Size	25,066 x 26,694
Coordinate system	WGS 84 (EPSG::4326)
Colors	3 bands, uint8
**Reconstruction parameters**	
Blending mode	Mosaic
Surface	DEM
Enable hole filling	Yes
Enable ghosting filter	No
Processing time	5 minutes 20 seconds
Memory usage	960.72 MB
Date created	2022:02:14 12:11:12
Software version	1.8.0.13794
File size	2.62 GB
**System**	
Software name	Agisoft Metashape Professional
Software version	1.8.0 build 13794
OS	Windows 64 bit
RAM	15.93 GB

CPU	AMD Ryzen 5 3600 6-Core Processor
GPU(s)	NVIDIA GeForce GTX 1660 SUPER
